# Bioinformatic and experimental identification and characterization of *Clostridioides difficile* lipoproteins as potential vaccine candidates

**DOI:** 10.3389/fimmu.2025.1650364

**Published:** 2025-12-17

**Authors:** Soumyadeep Chakraborty, Joshua Heuler, Shaohui Wang, Junling Qin, Adrit Roy, Lubem Nathanel Agbendeh, Anastasia Tomatsidou, Hyeun Bum Kim, Xingmin Sun

**Affiliations:** 1 Department of Molecular Medicine, Morsani College of Medicine, University of South Florida, Tampa, FL, United States; 2 Department of Biological Sciences, Fralin Life Sciences Institute, and Center for Emerging, Zoonotic, and Arthropod-borne Pathogens, Virginia Tech, Blacksburg, VA,, United States; 3 Department of Chemistry, University of South Florida, Tampa, FL, United States; 4 Department of Animal Biotechnology, Dankook University, Chenonan, Republic of Korea

**Keywords:** *Clostridioides difficile*, lipoprotein, vaccine, epitope analysis, mouse model, adhesion, colonization

## Abstract

**Introduction:**

Treatment options for *C. difficile* infection are limited, with very high rates of recurrence. Active vaccination provides an attractive opportunity to prevent *C. difficile* infection (CDI) and recurrence. In a search for potential surface-exposed antigens involved in *C. difficile* colonization, two putative lipoproteins, designated LP1 and LP2, were identified from *C. difficile* R20291.

**Methods:**

Lipoprotein sequences were aligned, analyzed, and evaluated for their immune properties. The antigenic characteristics of both LP1 and LP2 were assessed in silico and in a mouse model of immunization and CDI.

**Results:**

Multiple sequence alignments showed that the lipoprotein sequences were highly conserved among various ribotypes. *In silico* analysis predicted that LP1 and LP2 possess cytotoxic T-lymphocyte, helper T-lymphocyte, and B-cell epitopes with antigenic and immunogenic properties. Immune simulation provided insights into the ability of LP1 and LP2 to stimulate humoral and cellular immune responses. These properties were further examined in a mouse model of immunization and CDI. After three immunizations at 12-day intervals, significant amounts of IgG and IgA antibodies were detected in sera and feces. LP1 and LP2 immunizations provided mice with intermediate and higher levels of protection, respectively, against R20291 infection, and significantly reduced *C. difficile* spore and toxin levels in feces. Furthermore, anti-LP1 and anti-LP2 sera significantly inhibited adhesion of R20291 vegetative cells to HCT-8 gut epithelial cells.

**Discussion:**

These results indicate that both lipoproteins play a significant role in *C. difficile* adhesion and that LP1 and LP2 are promising immunogens for preventing *C. difficile* colonization.

## 1 Introduction

*Clostridioides difficile* is a Gram-positive, spore-forming, anaerobic bacterium, associated with severe diarrhea and colitis sometimes leading to death (1). Around half a million *C. difficile* infections are reported every year in the United States with very high rates of recurrence/relapse. CDI epidemics were also recorded in Europe (2), Asia (3) and Australia (4). *C. difficile* can produce two primary toxins TcdA and TcdB, which can disrupt gut epithelium (5) and a minor binary toxin CDT, which can disrupt cell cytoskeleton and induce protrusions on cell surface, facilitating the adhesion of *C. difficile* cells to the gut (6, 7).

Current standard CDI treatments rely on very few antibiotics, including vancomycin, fidaxomicin, or metronidazole, yet recurrence rates remain high. While fecal microbiota transplantation, defined microbial consortia, and live biotherapeutic products have shown promise in treating recurrent CDI (rCDI), efficacy remains variable and patient-dependent (8, 9). Active vaccination provides an attractive opportunity to prevent CDI and recurrence (10), but no vaccine against CDI has been licensed (11–13). Despite the need, the recent clinical trials in vaccine development targeting *C. difficile* toxins have been largely unsuccessful. Since *C. difficile* is an enteric pathogen, and persistent pathogen colonization is the root course of CDI and recurrence, effective vaccines should target not only toxins but also *C. difficile* colonization.

*C. difficile* surface molecules such as cell wall proteins (Cwp), S-layer proteins (SLP), microbial surface components have been reported as putative adhesion and colonization factors (14–16). ABC-transporter lipoprotein component in various pathogenic bacteria facilitated its adhesion to host cells (17, 18), among which one of the characterized lipoproteins from *Streptococcus pneumoniae* was protective against *Pneumococcal* colonization (19). Similarly, the surface-exposed lipoprotein CD0873 from *C. difficile* CD630 is an ABC transporter and is involved in adhesion and colonization to the host cell (20, 21). CD0873 was reported as a potent immunogen inducing strong secretory IgA immune response and protection against *C. difficile* colonization in the gut in mice. Another lipoprotein (CD1687) identified from *C. difficile* was involved in biofilm formation (22). In addition, lipoproteins have been reported to play important roles in *C. difficile* sporulation (23, 24).

Among the various roles of *C. difficile* lipoproteins this work was focused on the identification of potential immunogenic lipoproteins from hypervirulent strains of *C. difficile* R20291 with a goal to develop vaccine components against *C. difficile* colonization.

## 2 Results

### 2.1 Identification of putative LP1 and LP2 lipoprotein sequences in diverse *C. difficile* subtypes

The lipoprotein CD0873 from *C. difficile* 630 is an experimentally validated adhesin and potent immunogen as a potential vaccine component against CDI. *C. difficile* R20291 is a representative hypervirulent and epidemic RT027 strain in North America and Europe. Based on the DNA sequence of CD0873, we identified 2 homologues with 69% (LP1, Genebank accession: CBE02867.1) and 99% (LP2, Genebank accession: CBE02861.1) DNA sequence identities, respectively, from *C. difficile* R20291 genome.

We reasoned that LP1 and LP2 could be potential vaccine targets against CDI. To this end, we collected 150 C*. difficile* genomes representing major significant *C. difficile* ribotypes (RTs)/sequence types (STs)/clades and toxinotypes (25–29) (**Supplementary Table S1**) from either GenBank or the *Clostridioides difficile* genome database from Enterobase https://enterobase.warwick.ac.uk/) (30).

Out of 150 strains analyzed, only 13 did not encode full-length LP1. Eleven strains lacked a detectable LP1 sequence including VPI 10463 (RT003), AR-1091 (RT014), CD-17-01474 (RT027), DS 27638 (RT027), AR-1067 (RT027), AR-1076 (RT027), AR-1095 (RT027), CD-15-00010 (RT087), AR-1087 (RT106), AR-1093 (RT106), and TGH29 (ST1). Two strains, Z31 (RT009) and CD305 (RT023), encoded an LP1 sequence with a premature stop codon, suggesting that the resulting protein may be non-functional. While all the strains analyzed encoded LP2 protein sequence. Overall, the contrast between the widespread presence of LP2 in all examined *C. difficile* genomes and the absence of LP1 from several clinically relevant strains suggests that an LP2-based vaccine would provide better coverage and protection against diverse *C. difficile* types.

### 2.2 LP1 and LP2 relatedness correlates with host strain ribotype, but not toxinotype or clade

**Figure 1:**
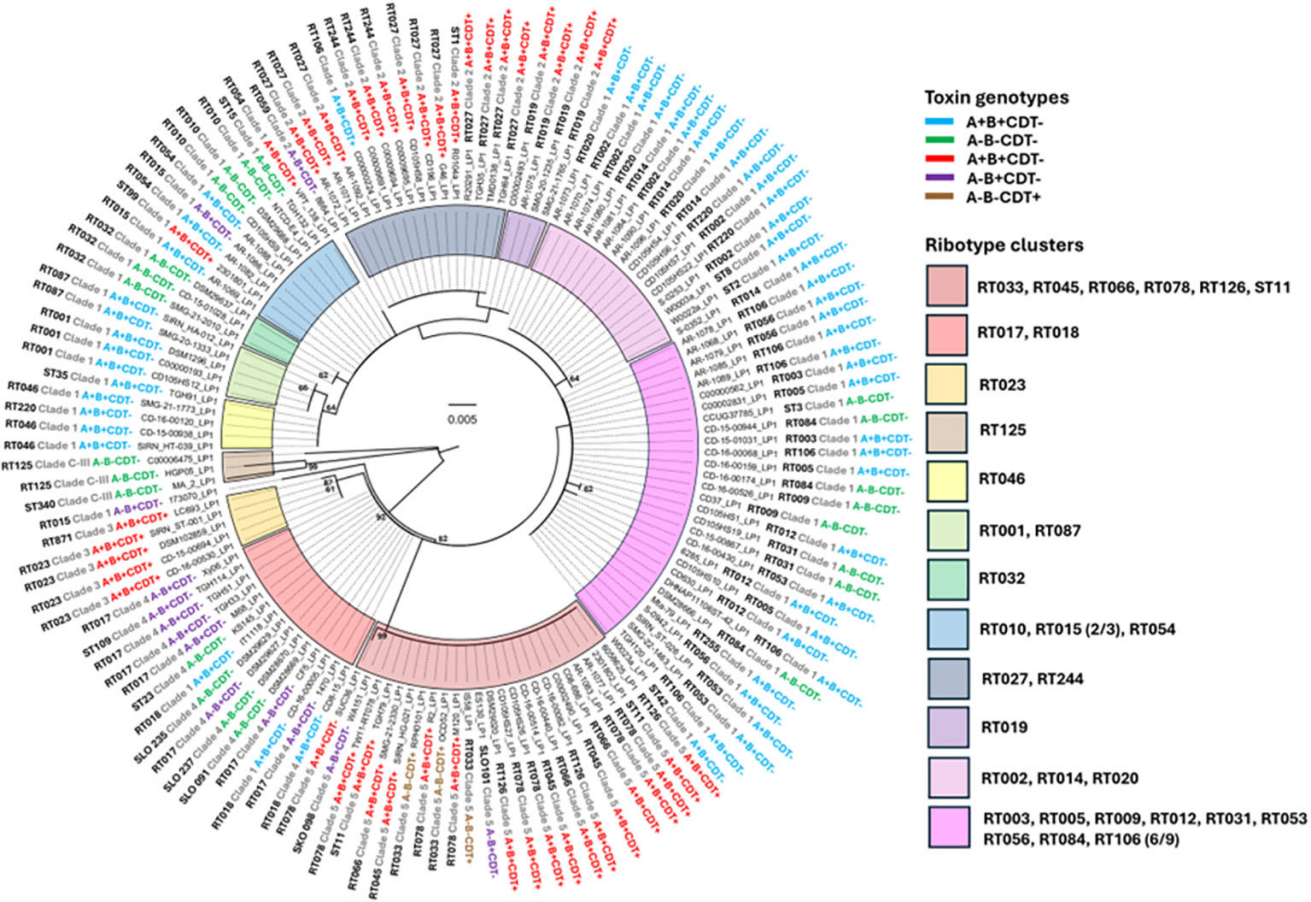
Phylogeny of LP1. Amino acid sequences of LP1 were aligned with the MUSCLE algorithm in MegaX before computing a Maximum Likelihood tree with 500 bootstrap replicates (bootstrap values >50 is displayed). Scale bars indicate 0.005 substitutions per site. The ribotype (or sequence type) and clade of each source strain is displayed adjacent to the strain name along with the toxin genotype as determined by nucleotide BLAST searches. Toxin genotype text is color coded and clusters of specific *C. difficile* ribotypes are shaded according to the legend on the right. For example, all representative strains of RT023 are in the orange cluster. Fractions next to ribotype names indicate that not all representative sequences of that ribotype are present in the cluster (e.g. two out of three RT015 sequences in the dataset are present in the light blue cluster).

**Figure 2:**
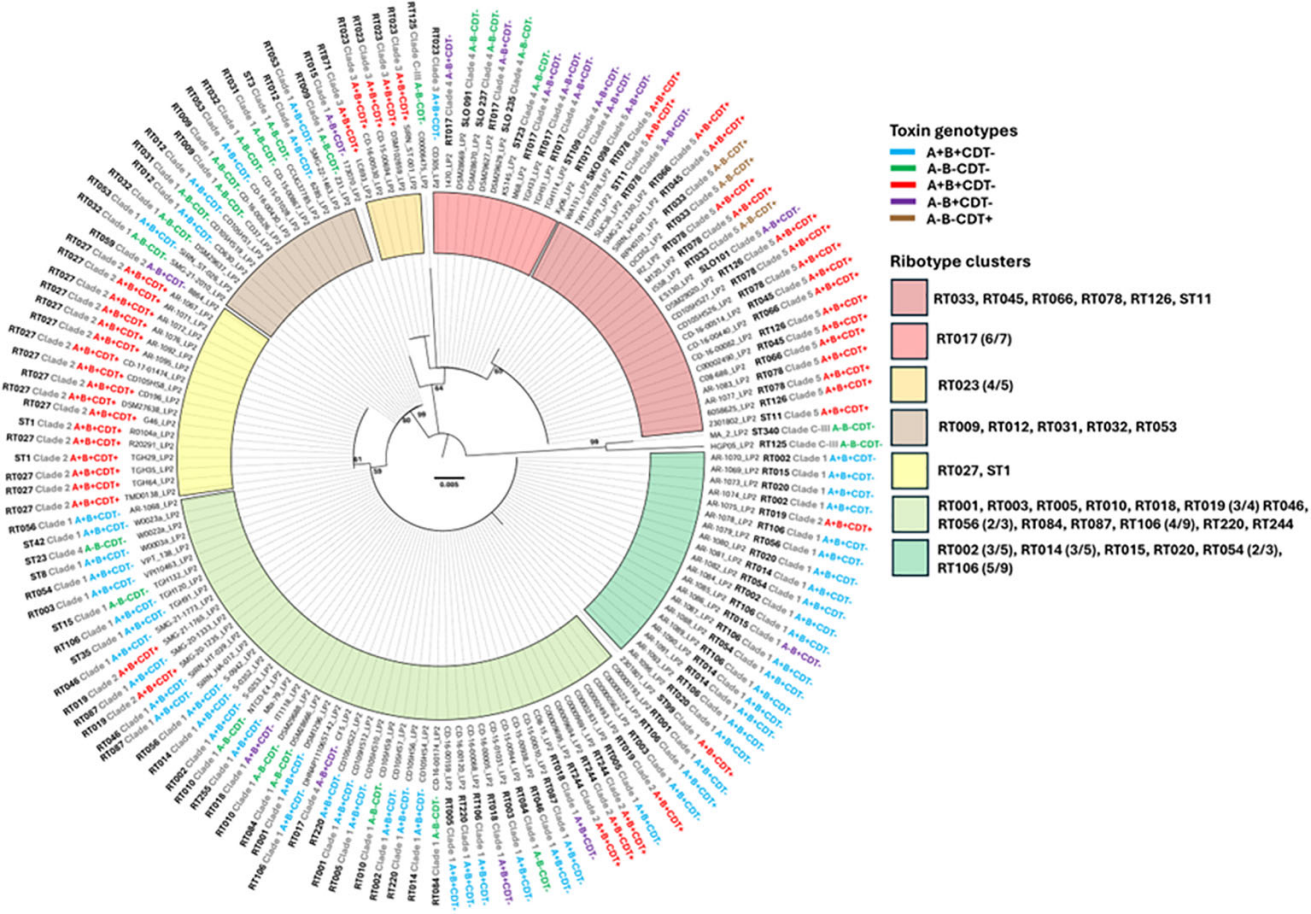
Phylogeny of LP2. Amino acid sequences of LP2 were aligned with the MUSCLE algorithm in MegaX before computing a Maximum Likelihood tree with 500 bootstrap replicates (bootstrap values >50 is displayed). Scale bars indicate 0.005 substitutions per site. The ribotype (or sequence type) and clade of each source strain is displayed adjacent to the strain name along with the toxin genotype as determined by nucleotide BLAST searches. Toxin genotype text is color coded and clusters of specific *C. difficile* ribotypes are shaded according to the legend on the right. For example, all representative strains of RT023 are in the orange cluster. Fractions next to ribotype names indicate that not all representative sequences of that ribotype are present in the cluster (e.g. six out of seven RT017 sequences in the dataset are present in the light red cluster).

It is critical for vaccine development that vaccine antigens should be conserved across various subtypes of *C. difficile*. To better understand how similar LP1 and LP2 sequences are among different *C. difficile* strains, we examined the phylogeny of LP1 and LP2 from multiple RTs/STs and toxinotypes (**Figures 1**, **2**). We classified 12 sequence clusters in the LP1 phylogenetic tree based on branching patterns (**Figure 1**). We observed that, for 32/33 RTs with more than one LP1 sequence in our dataset, a majority of the RT’s LP1 sequences could be found in a particular cluster of the tree. For example, *C. difficile* strains from subtypes RT033, RT045, RT066, RT078, RT126, and ST11 were all located in the dark red shaded cluster (**Figure 1**, **Table 1**), whereas the smaller purple shaded cluster featured exclusively RT019 LP1 sequences. While RT closely correlated with where a given LP1 sequence could be found on the tree, host strain toxiniotype did not correlate strongly. *C. difficile* strains with the same toxin genotype did not necessarily encode closely related LP1 sequences.

**Table 1:** LP1 and LP2 sequence representatives for homology analysis.

Cluster	Strain	Ribotype	Toxin genotype	Number of sequences
LP1/Figure 1				
Dark red	M120	RT078	A+B+CDT+	1
	SUC36	RT078	A+B+CDT+	2
Light red	M68	RT017	A-B+CDT-	3
Orange	CD-16-00530	RT023	A+B+CDT+	4
	SIRN_ST-001	RT023	A+B+CDT+	5
Singleton	173070	RT015	A-B+CDT-	6
Brown	MA_2	ST340	A+B+CDT+	7
	HGP05	RT125	A-B-CDT-	8
	C00006475	RT125	A-B-CDT-	9
Yellow	SIRN_HT-039	RT046	A+B+CDT-	10
Light green	CD105HS12	RT001	A+B+CDT-	11
Dark green	SMG-21-2010	RT032	A-B-CDT-	12
Light blue	AR-1069	RT015	A+B+CDT-	13
Singleton	8864	RT059	A-B+CDT-	14
Dark blue	R20291	RT027	A+B+CDT+	15
Purple	C00002493	RT019	A+B+CDT+	16
Pink	AR-1073	RT020	A+B+CDT-	17
Magenta	CD630	RT012	A+B+CDT-	18
	CD105HS19	RT031	A-B-CDT-	19
	CD-16-00430	RT053	A+B+CDT-	20
LP2/Figure 2				
Dark red	M120	RT078	A+B+CDT+	1
	WA151	SKO 098	A-B+CDT-	2
Light red	M68	RT017	A-B+CDT-	3
Singleton	C00006475	RT125	A-B-CDT-	4
Orange	SIRN_ST-001	RT023	A+B+CDT+	5
Singleton	173070	RT015	A-B+CDT-	6
Brown	Cd630	RT012	A+B+CDT-	7
Yellow	R20291	RT027	A+B+CDT+	8
Light green	AR-1068	RT056	A+B+CDT-	9
Singleton	2301801	ST99	A+B+CDT+	10
Dark green	AR-1096	RT020	A+B+CDT-	11
Singleton	HGP05	RT125	A-B-CDT-	12
Singleton	MA_2	ST340	A-B-CDT-	13

Similar findings were observed with LP2 phylogeny (**Figure 2**). Seven LP2 clusters were characterized on the phylogenetic tree compared to the twelve LP1 clusters, suggesting that LP2 sequences are less divergent than LP1. We observed that, for 33/34 RTs with more than one LP2 sequence in our dataset, a majority of each RT’s LP2 sequences could be found in a particular cluster of the tree. For example, all RT027 LP2 sequences are localized to the yellow shaded cluster (**Figure 2**, **Table 1**). Certain RTs even clustered the same way observed with LP1; RT033, RT045, RT066, RT078, RT126, ST11, RT017, and RT023 sequences were found in adjacent clusters in both trees. In some cases, certain strains are singletons in both trees, like 173070 (RT015). As was seen with LP1, *C. difficile* strains with the same toxiniotype did not necessarily encode closely related to LP2 either. Taken together, *C. difficile* RT correlates strongly with LP1 and LP2 similarity, whereas toxiniotypes do not correlate with the relatedness of LP1 or LP2.

### 2.3 LP1 and LP2 are well-conserved among various ribotyes and toxinotypes

**Figure 3:**
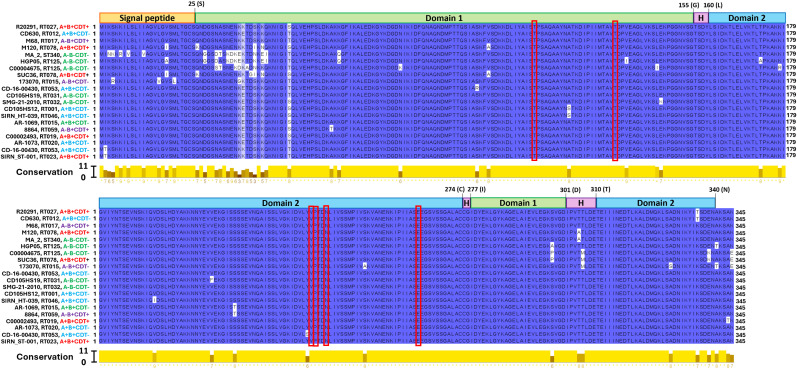
LP1 homology. Jalview software was used to illustrate MUSCLE alignments of LP1 amino acid sequences. The domain boundaries are illustrated and labelled as follows; signal peptide (1-25), domain 1 (26-155, 277-301), domain 2 (160-274, 310-340), and hinge region (“H”, 156-159, 275-276, 302-309). Conservation scores between 0 (no agreement) and 11 (identical amino acids) are shown for each amino acid position. Tyrosine binding amino acid residues are enclosed in red boxes. Strain ribotype, clade, and toxin genotype are mentioned to the right of the strain name.

To examine the homology of LP1 and LP2 sequences, we selected all non-identical sequences from the LP1 and LP2 phylogenetic trees and aligned them using the MUSCLE algorithm. Among the twenty LP1 sequences examined (**Figure 3**), we observed 49 non-identical residues (14.2% of the total 345 residues). When considering pairwise comparisons between all LP1 sequences in the dataset, sequences were 92.5%-100% identical. The LP1 homologues of CD630 and R20291were 99.1% identical. LP2 contained less non-identical amino acids compared with LP1 (27/340 total residues, or 7.9%). For pairwise comparisons, sequence identity ranged from 94.7-100% in the total LP2 dataset. CD630 and R20291, for example, encoded LP2 sequences that were 99.4% identical. These results suggest a high level of conservation between LP1 and LP2 sequences of different *C. difficile* strains.

Next, we analyzed how these mutations are distributed throughout the structures of the LP1 and LP2 proteins. R20291 LP1 and LP2 are 69% and 99%, respectively, similar to the *C. difficile* CD0873 lipoprotein, which has two domains connected by a hinge region (31). Although LP1 has less similarity to CD8073, it has a similar layout of a signal peptide followed by two domains connected by a hinge region according to the structures of other closely related LP1 sequences in AlphaFold (e.g. CD196 LP1, accession number AF-A0A0H3N134-F1-v4). According to the homology analysis the non-identical amino acid positions observed among LP1 sequences were highest in the signal peptide region (9/25 non-identical residues, or 36% of the region), domain 1 (25/155 non-identical residues or 16.2% of the region), domain 2 (10/146 non-identical residues, or 6.8%) and hinge region (2/15 non-identical residues or 13.3% of the region). While the LP2 sequences, non-identical amino acids observed in peptide region (5/25 non-identical residues, or 20% of the region), domain 1 (15/155 non-identical residues or 9.6% of the region), domain 2 (4/146 non-identical residues, or 2.7% of the region) and hinge region (3/15 non-identical residues or 20% of the region).

**Figure 4:**
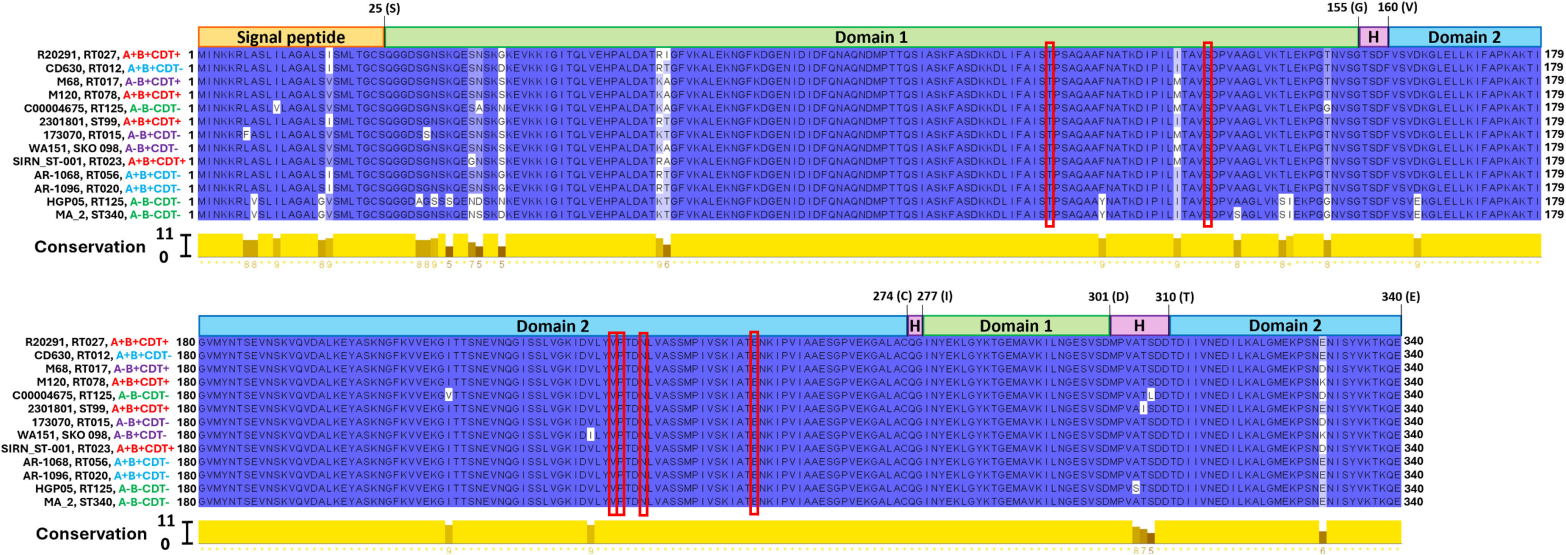
LP2 homology. Jalview software was used to illustrate MUSCLE alignments of LP2 amino acid sequences. The domain boundaries are illustrated and labelled as follows; signal peptide (1-25), domain 1 (26-155, 277-301), domain 2 (160-274, 310-340), and hinge region (“H”, 156-159, 275-276, 302-309). Conservation scores between 0 (no agreement) and 11 (identical amino acids) are shown for each amino acid position. Tyrosine binding amino acid residues are enclosed in red boxes. Strain ribotype, clade, and toxin genotype are mentioned to the right of the strain name.

Overall, LP2 showed more conservation amongst the sequences in our dataset, suggesting that LP2 could be a better target for *C. difficile* vaccination due to less variation between strains. However, both LP1 and LP2 exhibited little sequence variation within domain 1 or domain 2, with most variations occurring between the signal peptide and domain 1 instead. This suggests that most of the key structural components of LP1 and LP2 are highly conserved, making both lipoproteins potential vaccine targets. Much of the sequence variation observed in the multiple sequence alignment (**Figures 3**, **4**) are due to only a few strains such as 173070, MA_2, HGP05, and C00006475. The LP1 and LP2 sequences of these strains also appear genetically distinct in the phylogenetic trees (**Figures 1**, **2**), further demonstrating that these strains are outliers compared to the rest of the dataset. Taking together, most LP1 and LP2 sequences examined are highly conserved, with LP2 showing slightly higher conservation than LP1.

### 2.4 Both LP1 and LP2 are predicted to be antigenic based on epitope analysis with the latter being a potentially stronger immunogen

Epitope analysis of LP1 & LP2 sequence determined the presence of 3 and 6 potential Cytotoxic T-lymphocyte epitope in LP1 and LP2 sequence, respectively (**Table 2**). Helper T-lymphocyte epitope detected were 3 and 2 for LP1 and LP2, respectively (**Table 3**). Whereas 10 and 9 B-cell epitopes were identified in LP1 and LP2 sequences, respectively (**Table 4**). A closer look at the antigenicity values of the identified epitopes revealed that LP2 antigenicity values were higher than that of LP1. The entire protein LP1 and LP2 were identified as probable protective antigens with an antigenic value of 0.5139 and 0.5240, respectively, from Vaxijen 2.0. Both the proteins were identified as a probable immunogen with an immunogenic index of around 66% from Vaxijen 3.0.

**Table 2:** Listed below are all the Cytotoxic T-lymphocyte epitopes from LP1 & LP2.

Protein	Start position	Epitope	Combined score	Antigenicity	Toxicity	Allergenicity	Rank (%)	Best binging allele
LP1	116	AQAAYNATK	1.017	0.46	–	–	0.23	HLA-A*11:01
							0.38	HLA-A*30:01
							0.44	HLA-A*03:01
	125	DIPIIMTAV	1.0734	0.49	–	–	0.17	HLA-A*68:02
							0.64	HLA-A*26:01
							0.73	HLA-B*51:01
	298	VGDIPVTTL	0.7639	0.87	**-**	**-**	0.87	HLA-B*51:01
							1.1	HLA-B*07:02
							1.1	HLA-B*08:01
							1.2	HLA-A*02:06
							1.6	HLA-B*53:01
LP2	112	STPSAQAAF	0.7667	0.61	–	–	0.62	HLA-A*24:02
							0.68	HLA-B*15:01
							0.77	HLA-A*23:01
							0.82	HLA-B*35:01
							0.85	HLA-A*32:01
							1.4	HLA-B*58:01
							1.6	HLA-A*01:01
							1.7	HLA-A*30:02
							1.8	HLA-B*57:01
							2	HLA-A*68:02
	185	SEVNSKVQV	1.2484	1.39	–	–	0.18	HLA-B*44:03
							0.19	HLA-B*44:02
							0.1	HLA-B*40:01
	238	NLVASSMPI	0.9109	0.67	–	–	1.7	HLA-A*02:03
	270	ALACQGINY	1.5616	0.66	–	–	0.24	HLA-B*15:01
							0.28	HLA-A*30:02
							0.64	HLA-A*01:01
							1.2	HLA-A*26:01
							1.2	HLA-A*03:01
	275	GINYEKLGY	0.9098	0.95	–	–	0.2	HLA-A*30:02
							0.47	HLA-B*15:01
							0.82	HLA-A*01:01
							1.1	HLA-A*03:01
							1.3	HLA-A*26:01
							1.5	HLA-A*11:01
	295	GESVSDMPV	1.471	0.63	–	–	0.67	HLA-B*40:01

**Table 3:** Listed below are all the Helper T-lymphocyte epitopes from LP1 & LP2.

Protein	Start position	Epitope	Antigenicity	Toxicity	Allergenicity	IC50	Rank (%)	Best binding allele	IFN	IL4
LP1	108	IYAISTPSAQAAYNA	0.6	–	–	75.6	1.2	DRB1_1201	+	+
	317	TLKALDMQKLSADNI	0.59	–	–	49.7	1.2	DRB1_1201	+	+
				–	–	35.5	1.6	DRB4_0101		
	320	ALDMQKLSADNIKYI	0.49	–	–	7	1.3	DRB3_0301	+	+
LP2	106	DLIFAISTPSAQAAF	0.45	–	–	17.2	0.4	DRB1_0401	+	+
						5.2	0.01	DRB1_0404		
						14.5	1.8	DRB1_1001		
	200	ASKNGFKVVEKGITT	0.45	–	–	13.8	1.3	DRB1_1101	+	+

**Table 4:** Listed below are all the B-cell epitopes from LP1 & LP2.

Protein	Start position	Epitope	Score	Antigenicity	Allergenicity	Toxicity
LP1	69	LEDKGYKDGDNIKIDF	0.67	1.76	**-**	**-**
	85	QNAQNDMPTTQSIASK	0.78	0.6	**-**	**-**
	112	ISTPSAQAAYNATKDI	0.88	0.43	**-**	**-**
	140	AGLVKSLEKPGGNVSG	0.77	0.41	**-**	**-**
	148	KPGGNVSGTSDYLSID	0.78	1.58	**-**	**-**
	190	SKIQVDSLHDYAKKNN	0.8	0.55	**-**	**-**
	198	HDYAKKNNYEVVEKGI	0.65	0.63	**-**	**-**
	206	YEVVEKGISTSSEVNQ	0.79	0.72	**-**	**-**
	217	SEVNQAISSLVGKIDV	0.59	0.52	**-**	**-**
	328	SADNIKYIKSDENAKS	0.78	0.8	**-**	**-**
LP2	69	LEDKGYKDGDNIKIDF	0.67	1.76	–	–
	85	QNAQNDMPTTQSIASK	0.78	0.6	–	–
	112	ISTPSAQAAYNATKDI	0.88	0.43	–	–
	140	AGLVKSLEKPGGNVSG	0.77	0.41	–	–
	148	KPGGNVSGTSDYLSID	0.78	1.58	–	–
	190	SKIQVDSLHDYAKKNN	0.8	0.55	–	–
	198	HDYAKKNNYEVVEKGI	0.65	0.63	–	–
	206	YEVVEKGISTSSEVNQ	0.79	0.72	–	–
	217	SEVNQAISSLVGKIDV	0.59	0.52	–	–

### 2.5 Both LP1 and LP2 are predicted to be immunogenic based on immune simulation

**Figure 5:**
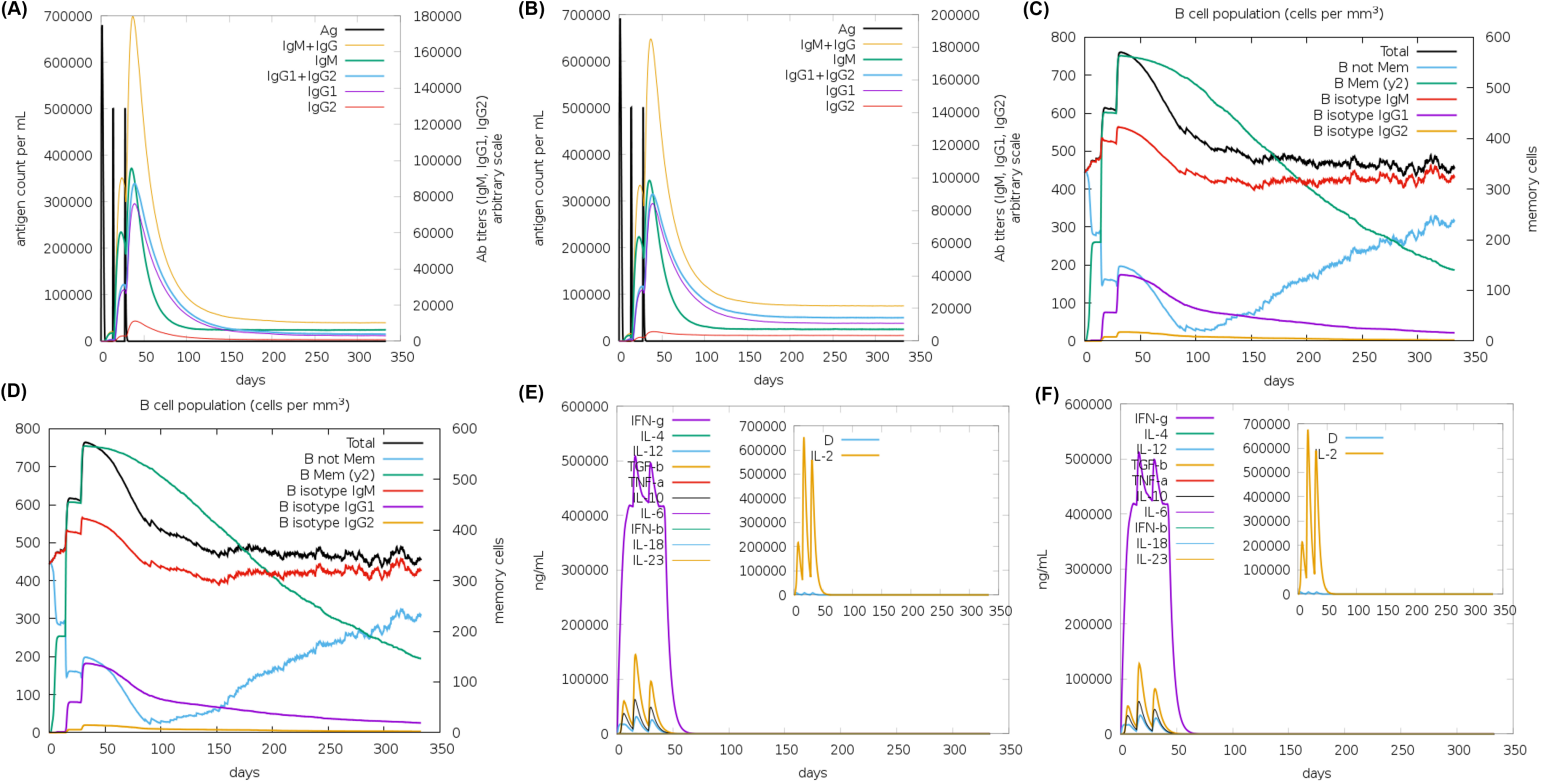
Immune simulation in CImmSim server, showing the antibody titers after 3 immunizations with the LP1 **(A)** and LP2 **(B)** 14 days apart shown in black spikes, the increase in the B-cell populations and class switching after immunization with LP1 **(C)** and LP2 **(D)**; the cytokine activation after immunization with LP1 **(E)** and LP2 **(F)**. ‘D’ in the insert plot indicates the potential danger levels to the immune system from immunogens LP1 **(E)** and LP2 **(F)**.

To predict immune responses against LP1 & LP2, we performed immune stimulation. Antibody titers both for LP1 and LP2 were significantly increased after second and third immunization as represented by the spike in IgG and IgM concentrations (**Figures 5A, B**) during the immune simulation in CImmSim (32) server. A classic example of immunoglobulin class switching was evident during the B-cell population analysis, where isotypes like IgM were higher after the first immunization, which was superseded by IgG1 and IgG2 isotypes after the second and third immunization (**Figures 5C, D**). It was also evident that LP1 and LP2 can induce balanced Th1 (IFN-Ƴ, IL-2 and TNF-α)/Th2 (IL-4) immune responses as indicted by cytokine activations (**Figures 5E, F**). Cytotoxic (T_C_) and Helper (T_H_) (**Supplementary Figure S1**) cell activation were indicated by their population-based analysis. Macrophages, dendritic cells and natural killer cells were also activated by the antigenic LP1 and LP2 (**Supplementary Figure S2**). In summary, both the antigens could elucidate humoral and cellular immune responses together with the development of corresponding memory cells.

### 2.6 Both LP1 and LP2 induce significant antibody responses in mice with LP2 being a stronger immunogen

**Figure 6:**
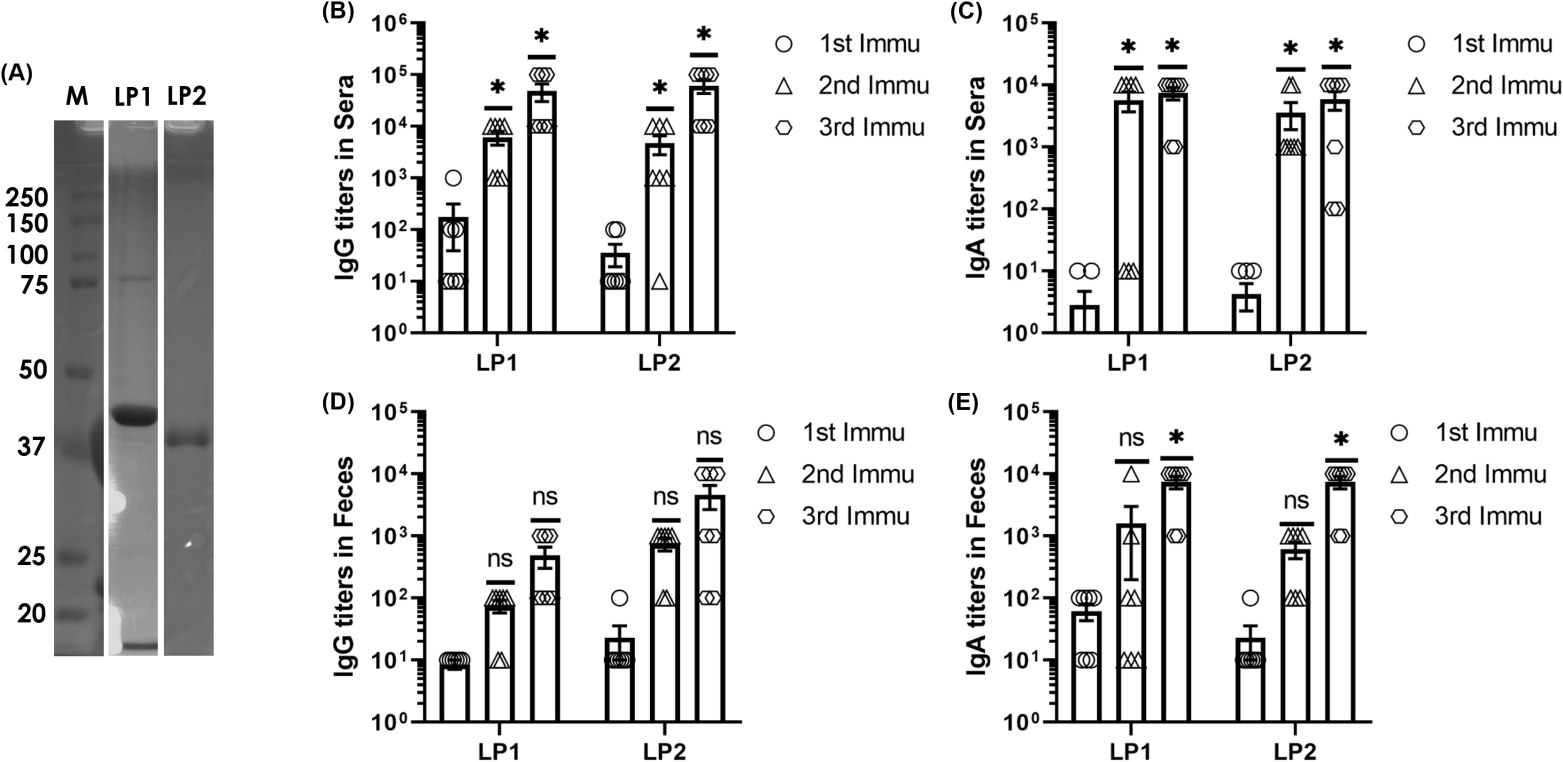
Ni-affinity chromatography-based protein purification of 38kDa Histag-LP1, and 37kDa Histag-LP2 **(A)**. Presence of anti-LP1 and anti-LP2 antibodies in the sera and feces of C57BL/6 mice (n=12) after immunization through intraperitoneal (i.p.) route thrice at an interval of 12 days. Anti-LP1 and anti-LP2 antibodies were measured by standard ELISA, which corresponds to **(B)** IgG titers and **(C)** IgA titers from sera; whereas **(D)** IgG titers and **(E)** IgA titers from feces. Experiments were performed in triplicate, and the data were represented as mean ± SD, (n=3; *P < 0.05; ns, no significance), compared to the antibody titers after first immunization.

To determine the immunogenicity *in vivo*, the recombinant LP1 and LP2 with a 6 x His-tag was expressed in *E. coli* BL21 and purified by Ni-affinity chromatography to greater than 95% purity (**Figure 6A**). Immunization of mice with 10 ug LP1 or LP2 mixed with Alum as an adjuvant via the intraperitoneal (i.p.) route induced significant levels of IgG and IgA antibodies in both sera (**Figures 6B, C**) and feces (**Figures 6D, E**) More specifically, LP 1 and LP2 induced comparable amounts of IgG antibodies in sera, and IgA antibodies in both sera and feces, while LP2 induced higher IgG antibody levels in feces.

**Figure 7:**
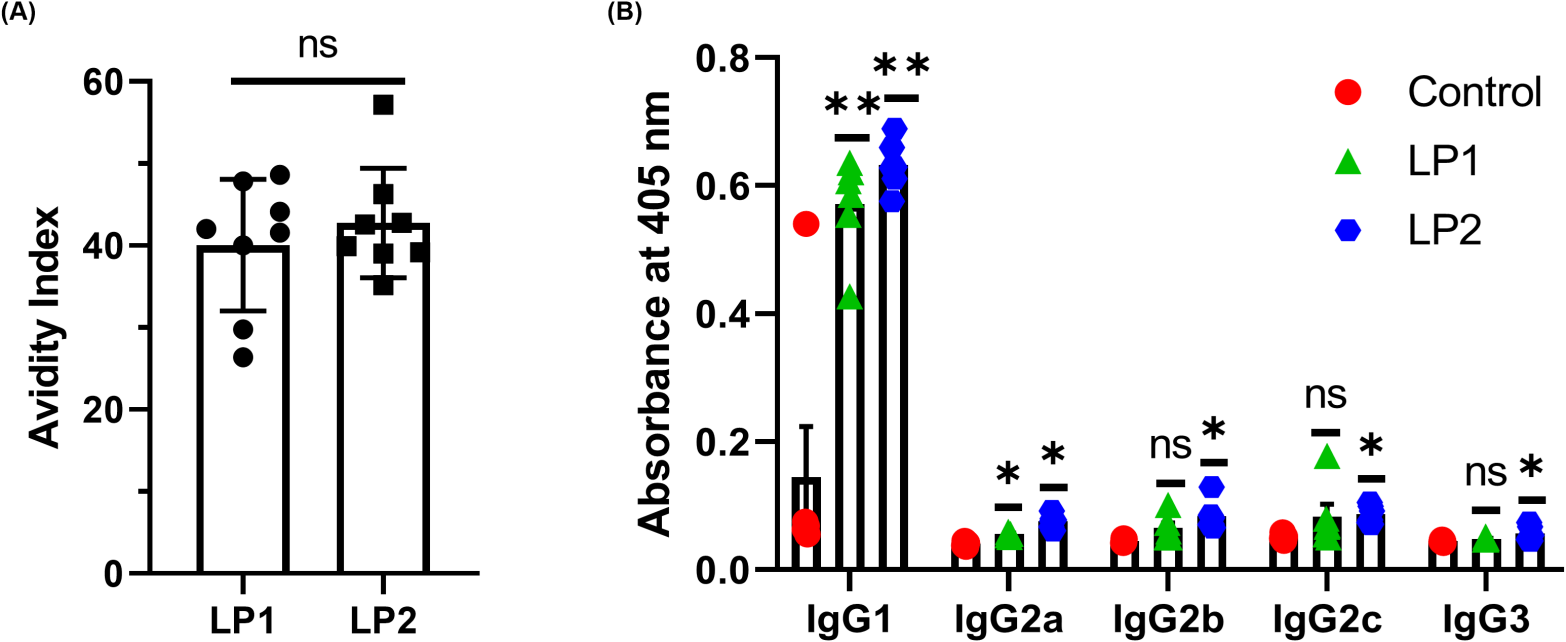
Anti-LP1/LP2 IgG isotypes and antibody avidities of sera from mice immunized with LP1 and LP2. Mice were immunized thrice with 10 µg/mouse of LP1 and LP2, followed by collection of serum samples. **(A)** Anti-LP1/LP2 antibody avidities. **(B)** The anti-LP1/LP2 IgG isotypes were measured from sera samples using standard ELISA techniques. The data were represented as mean ± SD (n=3; *P < 0.05; **P < 0.01; ns, no significance).

**Figure 8:**
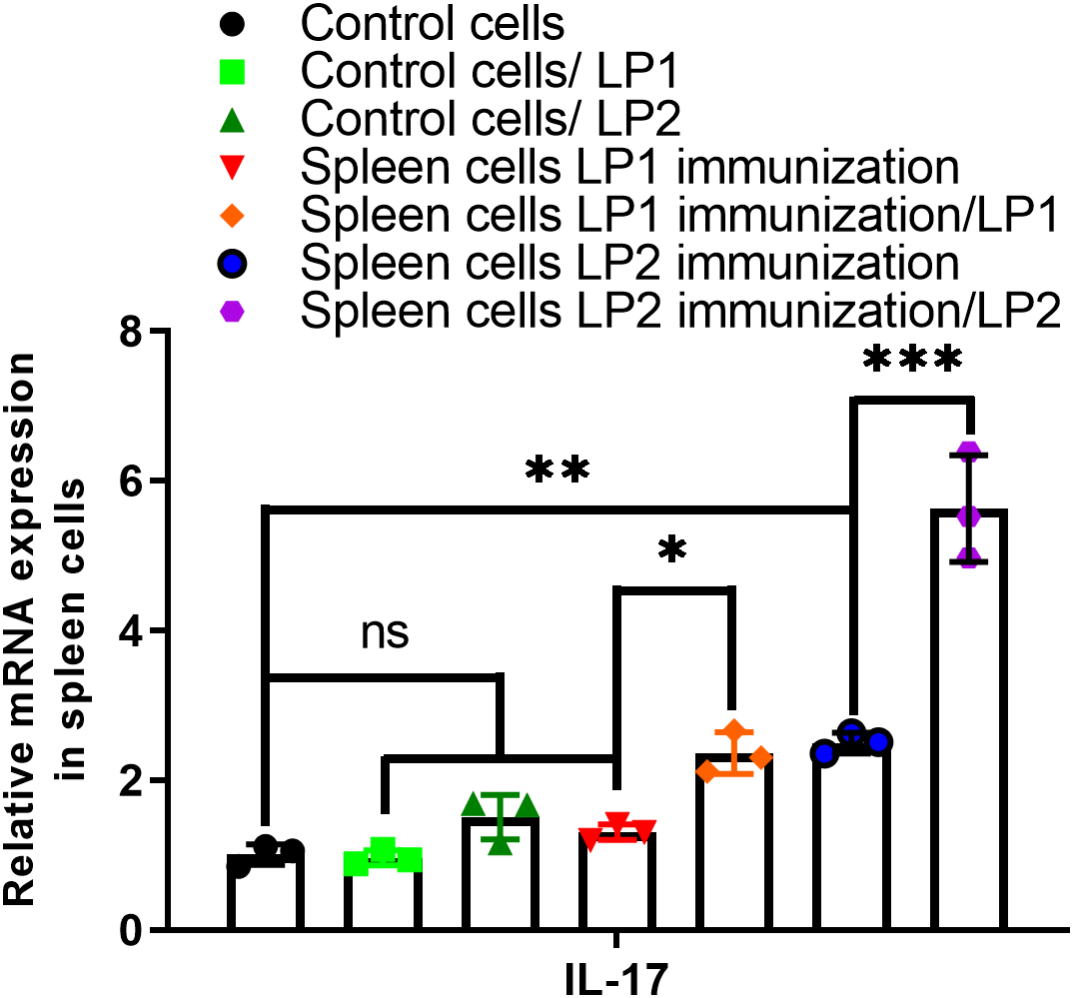
IL-17 expression in the immunized mouse-derived spleen cells, which were stimulated with protein LP1 or LP2. The y-axis indicates the expression ratio relative to GAPDH. Data were presented as mean relative expression ± standard error (n=3; *p<0.05; **p<0.01; ***p<0.001; ns, no significance).

Antibody avidity is the combined binding strength of all antigen-binding sites (paratopes) on an antibody interacting with their corresponding antigenic determinants (epitopes). Avidity reflects both intrinsic affinity (strength of a single antigen–antibody interaction) and the valency of the antibody (number of binding sites) and antigen. High avidity is a hallmark of antibody maturation and indicates better functional immunity. Avidity assays showed that sera from LP2-immunized mice demonstrated higher avidity though not significantly (*p* value = 0.4721) in comparison with LP1 (**Figure 7A**). In mice, IgG1 antibody is associated with Th2-like response, and IgG2a, IgG2b, IgG2c, and IgG3 antibodies are associated with Th1-like response. Both LP1 and LP2 with the adjuvant used primarily induced IgG1 antibodies, indicating a strong a Th2-like antibody response, while LP2 induced significantly stronger antibody responses of all IgG subtypes than LP1 (**Figure 7B**). When an antigen is administered (with or without adjuvant), IL-17–mediated responses can arise alongside or instead of Th1/Th2 responses, depending on the antigen type, adjuvant, and route of immunization. IL-17 promotes secretion of antimicrobial peptides and IgA at mucosal surfaces. RT-PCR showed that IL-17 was significantly increased in splenocytes of the LP2- not in the LP1- immunized mice (**Figure 8**).

**Figure 9:**
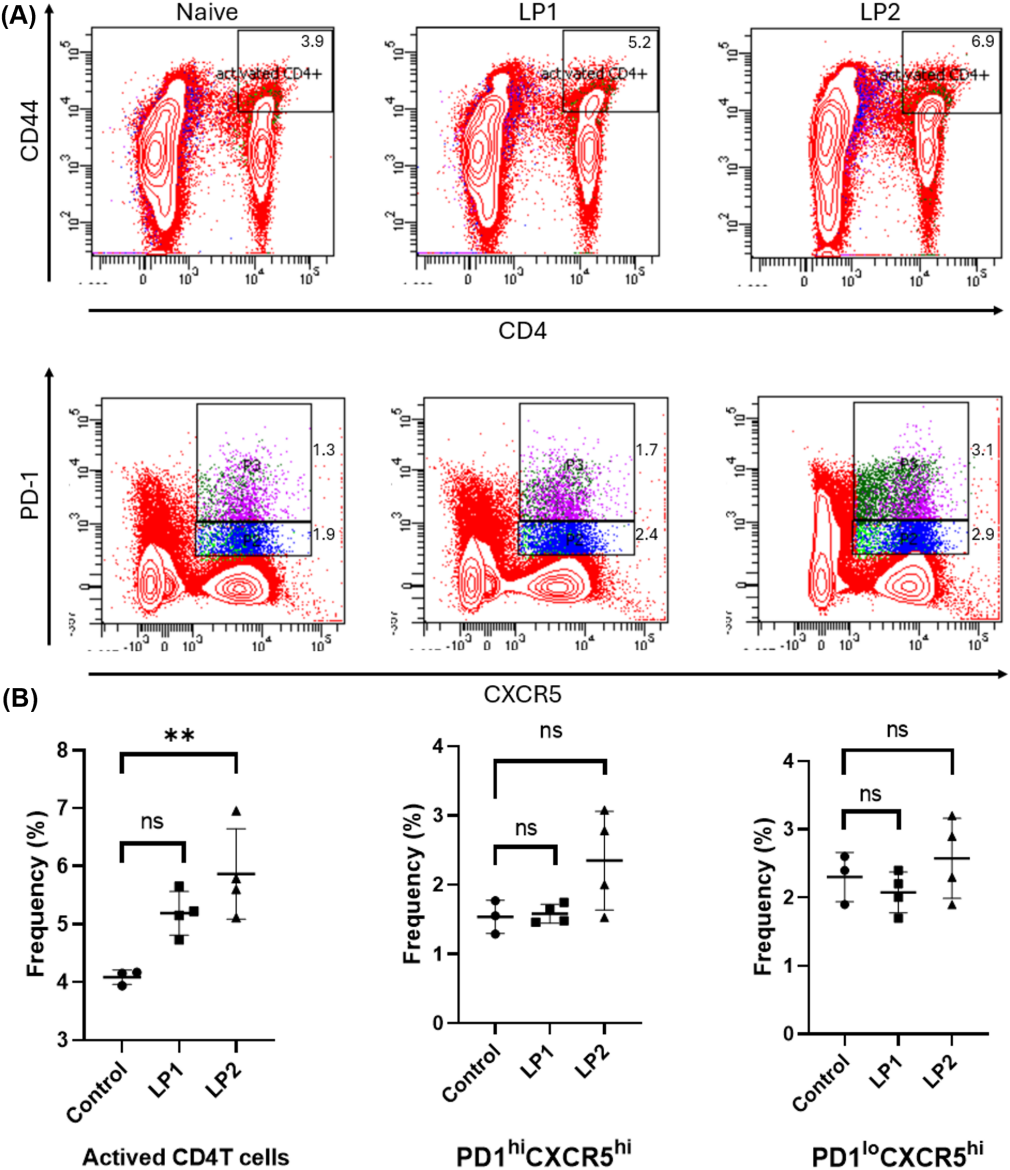
**(A)** Representative flow cytometry plots showing the gating strategy for identifying CD4^+^CD44^+^ activated T cells, PD-1^hi^CXCR5^hi^ and PD-1^lo^CXCR5^hi^ T follicular helper (Tfh) cell subsets within the lymphocyte population. Data is shown from naive, LP1, and LP2 immunized mice. **(B)** Quantification of the frequencies of CD4^+^CD44^+^ T cells (left), PD-1^hi^CXCR5^hi^ (middle), and PD-1^lo^CXCR5^hi^ (right) T follicular helper (Tfh) cell subsets. Each symbol represents an individual mouse. Statistical significance was determined using [insert test, e.g., one-way ANOVA with Tukey’s *post hoc* test], with significance denoted as follows: (n=3; *P < 0.05; **P < 0.01; ns, no significance).

**Figure 10:**
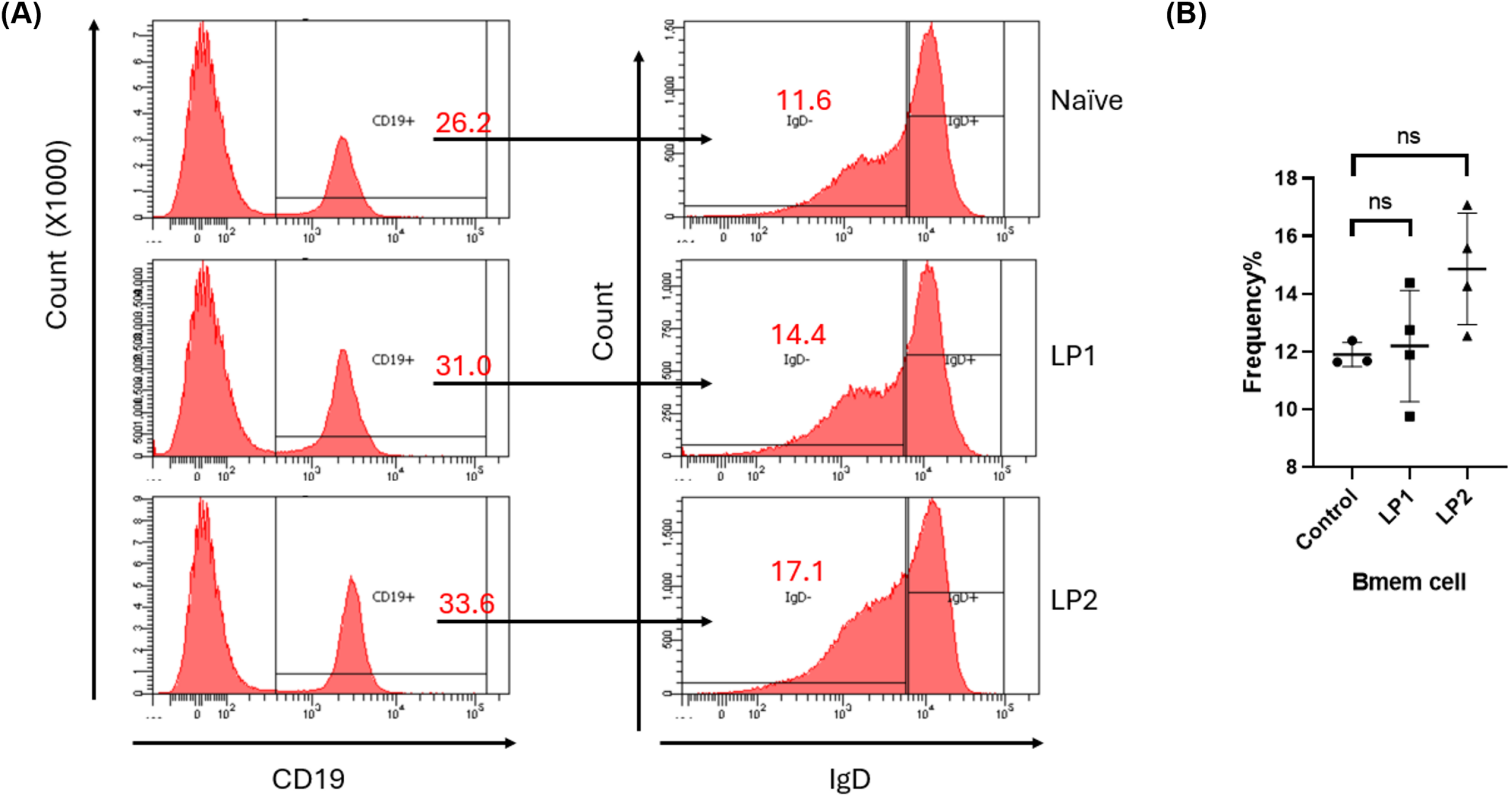
**(A)** CD19^+^ IgD^lo/-^ Bmem cells from naive, LP1, and LP2 immunized mice were identified. CD19^+^ cells were selected after gating on lymphocyte populations (left). IgD^lo/-^ were identified from the CD19^+^ cell population. **(B)** Quantification of the frequencies of Bmem cell. Statistical significance was determined using [insert test, e.g., one-way ANOVA with Tukey’s *post hoc* test], with significance denoted as follows: (n=3; *P < 0.05; ns: no significance).

**Figure 11:**
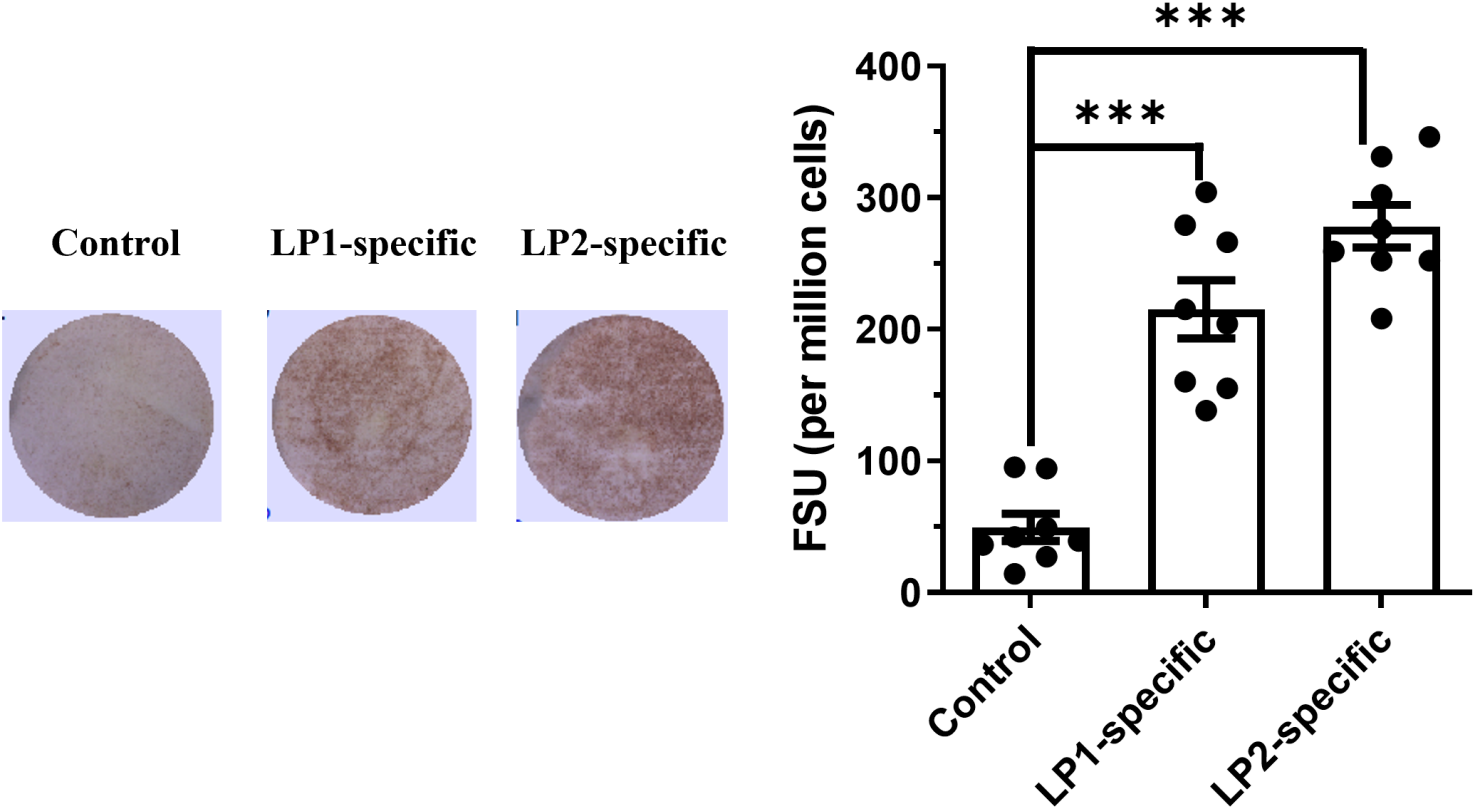
Representative images of ELISPOT plate wells with LP1 or LP2-specific spots (left) and numbers of LP1 or LP2-specific spots per million cells (right). ELISpot was used to compare antibody-secreting Bmem cells. Single cells were made from the bone marrow 3 weeks post-last immunization and activated with a B cell activator (R848/IL-2) for 48 h. All cells were then cultured in triplicate on LP1- or LP2-coated plates for an additional 24 h. Bone marrow cells from the control mice were used to measure baseline response. Spots were developed, counted, and plotted as spot-forming units (SFU) per million. Data were presented as mean relative weight ± standard error (n=3, ***p<0.001).

To further dissect the humoral immune responses elicited by LP1 and LP2 immunizations, we analyzed follicular helper T (Tfh) cells and memory B (Bmem) cells in the spleens of mice of day 14 post-immunization. Flow cytometry analyses showed that, compared to the control group, the LP2-immunized group exhibited a significant increase in activated helper T cells (CD4^^+^^ CD44^+^), while LP1 immunization only induced a trend of increase of these cells with no significance (*p* value = 0.06319) (**Figure 9**). Further analyses on Tfh subsets revealed LP2, but not LP1 induced a trend of expansion (with no significance) of either germinal center Tfh cells (GC-Tfh, CD4^+^ CD44^+^ CXCR5^hi^ PD-1^hi^) (*p* value = 0.9908) or non-germinal center Tfh cells (non-GC Tfh, CD4^+^ CD44^+^ CXCR5^hi^ PD-1^lo^) (*p* value = 0.7877) (**Figure 9**). In addition, LP 2, but not LP1, induced a trend of expansion (with no significance) of Bmem (CD19^+^ IgD^lo/-^) (*p* value = 0.1119) (**Figure 10**). Bone marrow is a key site for the persistence of Bmem and a long-lasting humoral immunity. Quantification of the Bmem in bone marrow cells using ELISpot assays revealed that LP2 induced a stronger Bmem response (**Figure 11**). Taken together, these data demonstrate that LP2 is a stronger immunogen than LP1.

### 2.7 Immunization with LP2 provides mice moderate but better protection than LP1 against *C. difficile* infection.

**Figure 12:**
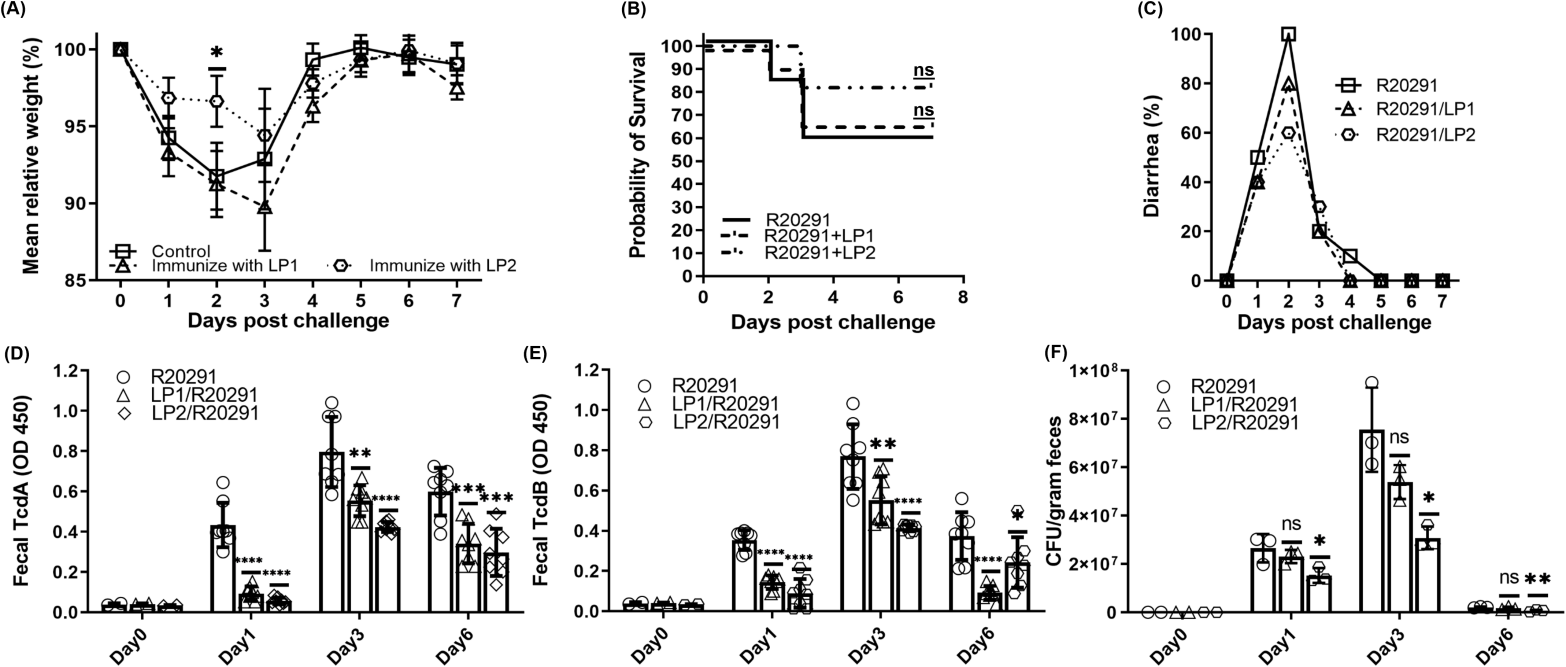
Protective immunity conferred by LP1/LP2 immunization against *C*. *difficile* infection. Immunized mice (n=12) were challenged with 10^6^ C. *difficile* R20291 spores. **(A)** Changes in the mean relative weight post infection. **(B)** Kaplan-Meier survival plot representing survival percentage post infection. **(C)** Occurrence of diarrhea among different groups post infection. Toxin levels in feces **(D)** TcdA and **(E)** TcdB. **(F)***C*. *difficile* R20291 spore levels in feces. The data were represented as mean ± SD. (n=3; *p < 0.05; **p < 0.01; ***p < 0.001; ****p < 0.0001; ns, no significance).

Mouse that was immunized three times with LP1 or LP2 at an interval of 14 days were subjected to challenge with 10^6^ spores of hypervirulent *C. difficile* R20291 strain. The control group that was not immunized appeared to lose an average of ca.10% of weight with a survival rate of 60%, and severe diarrhea in all mice were observed on day 2 of infection (**Figures 12A–C**). The overall weight loss for the group immunized with LP2 showed only ca. 5% reduction of weight, more than 80% survival and minimal diarrhea symptoms (**Figures 12A–C**). On the contrary LP1 didn’t seem to be very protective compared to LP2, the weight loss was like the control group with a survival of 65% and intermediate diarrhea symptoms (**Figures 12A–C**). Even if the LP1 immunization induced significant antibodies, it was not potent enough to induce significant protection against CDI in mice. LP2 is a more effective immunogen than LP1 against CDI.

LP1 and LP2 immunized mice showed comparatively but significantly less toxins in their feces, in comparison with the non-immunized group (**Figures 12D, E**). The amount of *C. difficile* R20291 spores recovered from the feces was highest on 3^rd^ day of infection (**Figure 12F**). Immunization of LP1 and LP2 both significantly reduced *C. difficile* spore levels in mouse feces; while the group immunized with LP2 showed lesser amounts of spores compared with the group immunized with LP1.

### 2.8 Both anti-LP1 and anti-LP2 sera inhibit the binding of *C. difficile* to human colonic cells

**Figure 13:**
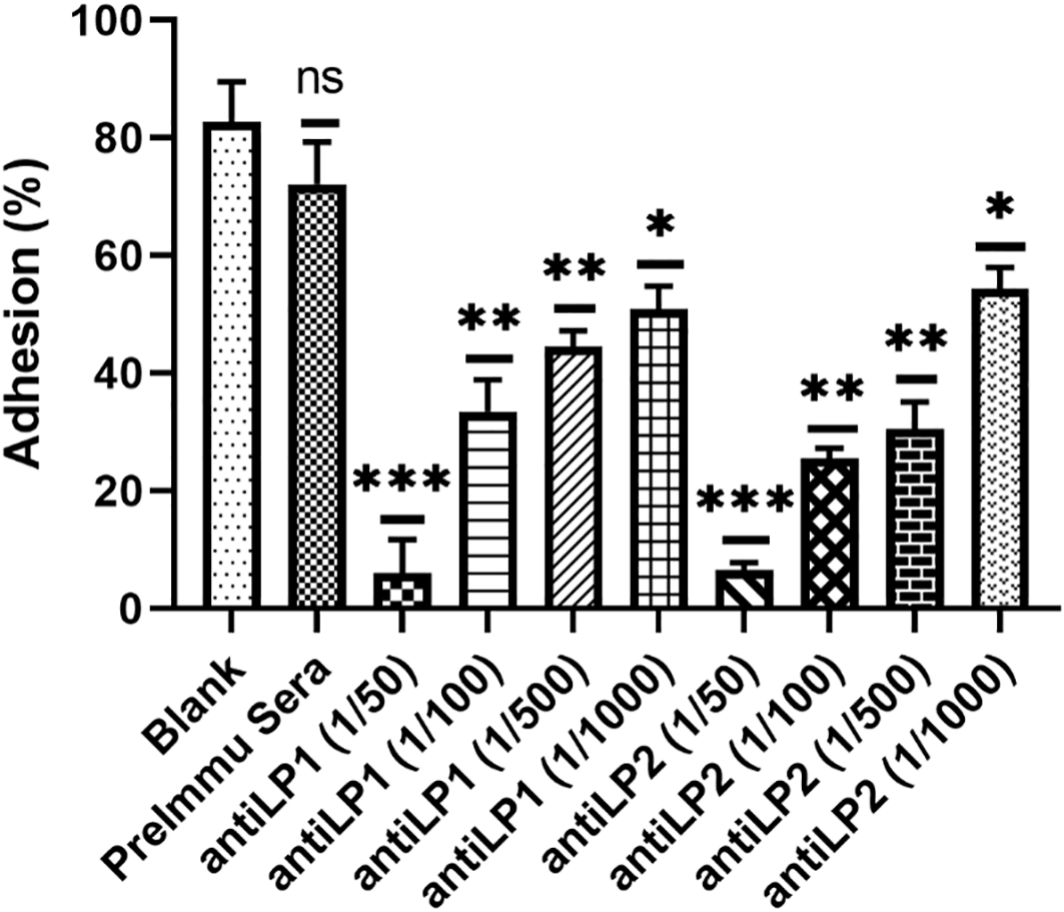
Adhesion of *C. difficile* R20291 vegetative cells to gut epithelial HCT8 were inhibited by different dilutions of (1/50, 1/100, 1/500 and 1/1000) anti-LP1 and anti-LP2 sera. The percent of *C. difficile* R20291 adhesion was calculated using the following formula: (initial CFU/mL – unbound CFU/mL)/initial CFU/mL. Experiments were performed in triplicates. The data were represented as mean ± SD. (n=3; *p < 0.05; **p < 0.01; ***p < 0.001; ns, no significance) (compared to the percentage adhesion from non-sera treatment).

Previously, *C. difficile* lipoprotein CD0873 was characterized as an adhesion/colonization factor (31). Our data showed that LP1 and LP2 immunizations significantly reduced *C. difficile* spores in feces of the immunized mice challenged with *C. difficile* spores (**Figure 13**). To investigate whether LP1 and LP2 are involved in *C. difficile* adhesion to intestinal cells, an *in-vitro* adhesion assay was performed. At dilutions of 1/50, 1/100, 1/500 and 1/1000 the anti-LP1 and anti-LP2 sera were able to significantly inhibit the attachment of *C. difficile* R20291 vegetative cells to HCT8 cells. The maximum adhesion inhibition was observed at 1/50 dilution (**Figure 13**). These data suggest that both LP1 and LP2 play an important role in *C. difficile* adhesion to the gut epithelium.

## 3 Discussion

Previously, lipoprotein CD0873 from strain *C. difficile* CD630, was characterized as a cell surface-exposed adhesion and colonization factor (21). CD0873 immunization protected mice against *C. difficile* infection in animal models (31).

In searching for homologues of CD0873 in the genome of the hypervirulent and epidemic *C. difficile* strain R20291 as a potential vaccine component against *C. difficile* colonization, two homologue lipoproteins (LP1 and LP2) were identified. Bioinformatic analyses showed that LP1 and LP2 among major *C. difficile* ribotypes/clades and toxinotypes were quite conserved and contained the signal peptide cleavage site after cysteine (C25) residue as reported previously (21). The crystal structure of CD0873 (PDB ID: 6HNK & 6HNI) showed the presence of two domains connected by a loop in α-β-α sandwich arrangement, the two domains come closer to bind a tyrosine molecule with corresponding amino acids residues T114, S135/T135, D158, V235, P236, N239 and E263 (**Figures 2**, **3**) (31). Sequence alignments of the lipoprotein sequences from the representative RT/Clade/ST strains showed the presence of the conserved Tyrosine binding amino acid residues, with only one exception at residue position 135, where a conserved Threonine (T) residue was identified in LP1 while there was a conserved Serine (S) residue in LP2. The signal peptide followed by the three domains among these lipoproteins were conserved, where signal peptide (1-25) domain 1 (26-155, 277-301), domain 2 (160-274, 310-340) and hinge region (156-159, 275-276, 302-309) showed conserved starting and ending sequences.

The detailed phylogeny analysis shows that *C. difficile* ribotype correlates strongly with LP1 and LP2 similarity, whereas toxin genotypes do not correlate with the relatedness of LP1 or LP2. Further homology analysis showed that both LP1 and LP2 were highly conserved, with LP2 being more conserved compared to LP1 although the overall sequence variation among the domains was not significantly high. This suggested that LP2 could be a better target for *C. difficile* vaccination due to less variation among different strains.

The epitope analysis of LP1 and LP2 revealed the presence of more potential CTL epitopes with higher antigenicity scores in LP2, whereas the number of HTL epitopes and their antigenicity scores were very similar. On the contrary, the number of B cell epitopes in LP1 and LP2 were similar; but a further antigenicity score analysis revealed that LP2 epitopes seem to be more antigenic than LP1. Therefore, the overall epitope analyses predicted that LP2 could induce stronger immune response than LP1. Further immune simulation showed that both LP1 and LP2 proteins were capable of significantly stimulating humoral and cellular immune responses. The immune simulation after third immunization illustrated that the antibody titers reached a concentration of around 10^5^, which is quite comparable to the experimental values achieved for various previously characterized vaccine candidates in mouse model (33–35).

Mice immunized with LP1 and LP2 showed a significant increase in IgG and IgA production after 3^rd^ immunization via intraperitoneal (i.p.) route. A significant increase was also observed in the secretory (s)IgA production detected in feces after immunization. Similar observations on increased sIgA levels after immunization with *C. difficile* lipoprotein were previously reported (31). The detection of higher level of IL-17 in LP2 immunized mice clearly indicated that it is a more potent antigen. Activation of helper T cells and memory B cells after immunization with LP1 or LP2 were detected in mouse splenocytes. The activation of memory B cells (Bmem) after immunization was further validated by ELISpot assays of bone marrow cells from the immunized mice. All these observations validated the immune simulation studies, which predicted the activation of helper T cells and memory B cells after administration of LP1 and LP2 antigens.

Immunizations with LP1 and LP2 conferred intermediate and higher protection, respectively, against CDI, which was also supported by the analysis of *C. difficile* toxin and spore levels in feces of the infected mice. The toxins (TcdA and TcdB) levels and *C. difficile* spores detected after CDI from LP1 and LP2 immunized mice were considerably low during the day 1, 3 and 6 post-challenges, indicating that both the immunogens could induce immune protection against *C. difficile* colonization and toxin-caused damages.

Lipoproteins are located on the cell surface of infectious bacteria and mainly involved in cell adhesion (17, 18). Further *in-vitro* adhesion assay showed that the LP1 and LP2 sera from immunized mice significantly inhibited the attachment of *C. difficile* to the HCT8 gut epithelium cells, which proved that the anti-LP1 and anti-LP2 sera raised after immunization can attach to *C*. *difficile* cells and inhibit their adhesion to epithelial cells. These observations confirmed that both LP1 and LP1 lipoproteins were protective against CDI hindering the *C. difficile* cell adhesion. Our data is also in agreement with previous reports that surface displayed lipoproteins are mainly involved in cell adhesion (17, 18).

## 4 Conclusion

LP1 and LP2 from *C. difficile* R20291 strain are potential immunogens that could stimulate humoral and cellular immune responses, thus protecting animals from CDI by reducing and hindering *C. difficile* cell adhesion and toxin production. Both LP1 and LP2 lipoproteins can be used together with other potential immunogens (*C. difficile* surface proteins, toxin fragments or spores coat proteins) for development of fusion immunogens leading towards a protective vaccine against CDI, with LP2 being a more potent immunogen.

## 5 Materials and method

### 5.1 Identification of *C. difficile* lipoprotein sequences

*C. difficile* strains were screened from available databases and previous studies (**Supplementary Table S1**), to develop subsets based on different major ribotypes/clades and toxinotypes. The genomes of the selected strains were retrieved from either NCBI GenBank (https://www.ncbi.nlm.nih.gov/genbank/) or the *Clostridioides difficile* genome database from Enterobase (https://enterobase.warwick.ac.uk/) (36). Clade information for each genome was determined by a combination of prior literature and a MLST search on the PubMLST database (https://pubmlst.org/bigsdb?db=pubmlst_cdifficile_seqdef&page=sequenceQuery) (37). CD0873–340 aa tyrosine ABC transporter substrate-binding lipoprotein sequences (Protein ID: WP_003437194.1) from *C. difficile* CD630 was used as a reference sequence. LP1 and LP2 amino acid sequences were mined from each genome, any sequences with a premature stop codon were excluded from further analysis.

### 5.2 Sequence alignment and phylogeny analysis of lipoprotein sequences from *C. difficile*

Multiple sequence alignment of the LP1 and LP2 sequences from *C. difficile* were performed using MUSCLE algorithm in MegaX (ver 11.0.13) (38) with the default settings. The maximum likelihood phylogenetic tree was constructed using 500 bootstrap replicates, using MegaX. The tree was visualized using the FigTree (ver 1.4.4) (https://tree.bio.ed.ac.uk/software/figtree/). The cluster pattern from these phylogenetic trees guided the selection of non-identical LP1 and LP2 sequences for domain analysis. The selected sequences were re-aligned using MUSCLE algorithm (ver 3.8.31) in the MPI Bioinformatics Toolkit Server (https://toolkit.tuebingen.mpg.de/tools/muscle) (39, 40) and finally viewed in Jalview (ver 2.11.4.1) (41).

### 5.3 Epitope analysis based LP1 and LP2 sequences

LP1 (345 aa, Genebank accession: CBE02867.1) and LP2 (340 aa, Genebank accession: CBE02861.1) sequences from *C. difficile* R20291 were subjected to epitope analysis by determination of Cytotoxic T-lymphocyte (CTL) epitopes by NetCTL 1.2 (https://services.healthtech.dtu.dk/services/NetCTL-1.2/) (42) with a threshold of 0.75, whereas the Helper T-lymphocytes (HTL) were determined by NetMHCII- 2.3 (https://services.healthtech.dtu.dk/services/NetMHCII-2.3/) (43) using a threshold of ≤ 2%, and the B cells epitopes were predicted with ABCpred (https://webs.iiitd.edu.in/raghava/abcpred/) (44). The antigenicity index of the entire protein LP1 and LP2, as well as the individual epitopes were determined using Vaxijen 2.0 (https://www.ddg-pharmfac.net/vaxijen/VaxiJen/VaxiJen.html) (45) with a threshold of 0.5. Vaxijen 3.0 (https://www.ddg-pharmfac.net/vaxijen3/home/) (46) helped us to analyze the immunogenicity index of LP1 and LP2. The individual epitopes were analyzed with Class 1 Immunogenicity (http://tools.iedb.org/immunogenicity/) (47) tool to understand their immunogenic properties, the threshold used was >0. Allergenicity and toxicity of the predicted epitopes were ascertained by AllerTOP v. 2.0 (https://www.ddg-pharmfac.net/AllerTOP/) (48) and ToxinPred (https://webs.iiitd.edu.in/raghava/toxinpred/algo.php) (49), respectively. The epitopes binding to the corresponding MHC Class I & Class II alleles were identified from TepiTool (http://tools.iedb.org/tepitool/) (50) on the IEDB server database, a threshold of ≤ 2% was used.

### 5.4 Immune simulation of LP1 and LP2 sequences

Identification of LP1 and LP2 to elicit immune response and confer protective immunity to the host was important. Hence both these proteins were subjected to an immune simulation in C-ImmSim server (https://kraken.iac.rm.cnr.it/C-IMMSIM) (32). The immunization regime used for the simulation included the injection of 1, 000 vaccine unit with 2-week interval between each immunization. The whole simulation process ran for 240 time-steps with injection time points set at 1, 42 and 84. The most frequently occurring MHC Class I and Class II alleles (A2602, A0101, B5301, B5101, DRB1_0404, DRB1_0101) identified during the epitope analysis from LP1 and LP2 were used in the simulation.

### 5.5 Expression and purification of LP1 and LP2

The LP1 (345 aa) and LP2 (340 aa) sequence was amplified from the genomic DNA of *C. difficile* R20291 using primer set in **Table 5**. BamHI and XhoI restriction sites were used to clone the amplified LP1 and LP2 gene into pET28a (+) vector. The cloned genes were expressed in *E. coli* BL21(DE3) cells after induction with Isopropyl β-D-1-thiogalactopyranoside (IPTG). Larger cultures of LP1 and LP2 were grown for purification of the protein by immobilized metal affinity chromatography (IMAC). Nickel-nitrilotriacetic acid (NTA) affinity columns were used for His-tag purification of the proteins with a 95% purity (**Supplementary Figure S1**). The purified protein was concentrated and the buffer exchanged with phosphate buffer saline (PBS) which made it suitable for injection to the mice during immunization.

**Table 5:** Primer sequences used for amplifying LP1 and LP2 genes from the genomic DNA of *C. difficile* R20291.

Gene	Forward primer	Reverse primer
LP1	5’-GCATATGGATCCATGCTTACTGGATGTTCTCAAAATGATGGCTCCAAT-3’	5’-GCGCGCCTCGAGTTATTTTGCAGATTTTGCATTTTCATCTGA-3’
LP2	5’-ATATATGGATCCATGCTTACAGGGTGTTCACAAGGAGGAGACTCTGGT-3’	5’-GCGCGCGCCTCGAGCTATTCTTGTTTAGTCTTTACATAAGAAAT-3’

### 5.6 *C. difficile* spore preparation

Freshly prepared 70:30 sporulation medium (51) was used for generation of *C. difficile* spores. The spores were scrapped from the surface of the 70:30 plates after 4–5 days of growth at 37°C in the anaerobic chamber. The spore suspension was pelleted at 10, 000 g for 20 mins and washed 5 times with sterile distilled water. The washed spores were resuspended in 5 ml of sterile water, which was then layered on to 10 ml of 50% sucrose. The liquids containing the cell debris were removed after centrifugation at 15, 000g for 15 min. The spores were again washed 5 times with sterile water to remove any excess sucrose and finally resuspended in 200 µl of sterile water. The spore purification protocol was adapted with specific modification to a previously published method (52). The purified spores were counted on TCCFA or BHI plates after serial dilutions from the spore stock. Requisite among of spore stock was used for dilution in PBS before immunizing the mice.

### 5.7 Immunization and *C. difficile* infection in mouse model

All studies followed the Guide for the Care and Use of Laboratory Animals of the National Institutes of Health and were approved by the Institutes Animal Care and Use Committee (IACUC) at the University of South Florida. C57BL/6 mice were purchased from Charles River Laboratories. Both Male and Female C57/BL6 mice were housed in separate cages in a specific pathogen free (SPF) environment under semi-natural light cycle of 14 hours light and 10 hours dark. The mouse was housed in an infection room during the immunization and *C. difficile* infection process. Mice (n=12, Male-6 and Female-6) were immunized 3 times in an interval of 12 days with 10 µg in 100 µl of purified LP1 and LP2 proteins for each mouse via intraperitoneal (i.p.) route. Sera was collected on the 12th day after each immunization, where anti-LP1 and anti-LP2 antibodies were detected by ELISA. After 12 days of third immunization the immunized mice and the control group were administered with antibiotic mix water containing ampicillin (200mg/kg), clostin (4.2 mg/kg), gentamycin (3.5 mg/kg), kanamycin (40mg/kg), metronidazole (21.5 mg/kg) and vancomycin (4.5 mg/kg) for 5 days, followed by 2 days of sterile water. Clindamycin (10mg/kg) single dose was administered via intraperitoneal (i.p.) injection before challenge with 100 µl of 10^6^ C*. difficile* R20291 spores via oral gavage as mentioned previously (53). All the mice were monitored daily for weight loss, diarrhea, survival and any other physical symptoms after the infection. Diarrhea was observed as a symptom of wet tail, loose or watery feces. Mice with >20% weight loss and mice died after infection were considered as death and included in the survival plot.

### 5.8 Quantification of anti-LP1 and anti-LP2 by ELISA

ELISA for detection of anti-LP1 and anti-LP2 in sera and feces of immunized mice were performed in 96 well plates. Each well was coated with 100µl of 1 µg/ml of LP1 or LP2 and incubated overnight at 4°C. The plates were washed with 200 µl of blocking buffer (5% dry milk in PBS) and incubated for 2 hours at 4°C. After washing, 100 µl of 10-fold diluted sera or fecal samples were pipetted into each well and incubated at room temperature for 1.5 hours. The plates were again washed and secondary antibody 100 µl of IgG-HRP (1:3000) and IgA-HRP (1:3000) were added to each well and incubated at room temperature for 1 hour. The substrate for detection (TMB 50µl) were added to each well and incubated in dark for 30 min. The reaction was stopped by adding 25 µl of H_2_SO_4_ to each well and the plate was immediately read for corresponding OD values at 450 nm in a BioGene ^®^ plate reader. The anti-IgG and anti-IgA titers were determined as the function of the degree of dilution where the OD_450_ values in the sample were 2-fold of the values in non-immunized mice.

### 5.9 Avidity assay

Avidity of antibodies towards LP1 and LP2 antigens were determined using urea disruption ELISA method. The plate was coated with 5µg/mL of LP1 and LP2 separately and incubated overnight at 4°C. The washing and blocking steps are similar to the ELISA method mentioned in section 5.8. Serum samples were tested after fourfold dilution in two separate rows, with dilution ranging from 1 to 12.5. After incubation in room temperature for 1.5 hours, row 1 was treated with 200 µl of dissociation buffer (PBST containing 6 M urea) and row 2 was treated with 200 µl of PBST. The plates were incubated at 37°C with shaking for 30 min. The plates were washed and incubated with secondary antibodies, followed by their development and reading as mentioned earlier in Section 5.8. The percentage avidity was expressed as Avidity Index calculated by the following formula: Avidity Index = {(average absorbance of urea-treated sample)/(average absorbance of non-treated sample) x 100}.

### 5.10 Detection of anti-LP1 and anti-LP2 IgG isotypes

IgG isotypes were determined against sera from LP1 and LP2 immunized mice by standard ELISA techniques mentioned earlier in Section 5.8. Biotinylated anti-mouse IgG isotype antibodies IgG1, IgG2a, IgG2b and IgG3 anti-LP1 and anti-LP2 were used for the process.

### 5.11 Real-time PCR for determination of cytokines in immunized sera

Spleens were procured from mice previously immunized with LP1, LP2, or a control substance, 21 days following the third and final administration. To generate a single-cell suspension, splenocytes were dissociated through sequential filtration using 70 µm and 40 µm cell strainers, facilitated by syringe plungers. Following enumeration, cells were cultured in RPMI 1640 medium (Thermofisher, USA) supplemented with 10% and maintained at 37°C in a 5% CO_2_ atmosphere. The cells were subsequently stimulated (pulsed) overnight with 10 µg/mL of either LP1 or LP2. Post-incubation, the cells were harvested for real-time Polymerase Chain Reaction (PCR) analysis, with GAPDH serving as the internal standardization reference gene.

### 5.12 Flow cytometry to study immunized mice splenocytes

Single-cell suspensions were prepared from spleens by mechanical disruption, followed by red blood cell lysis using ACK lysing buffer (Gibco). Cells were resuspended in RPMI 1640 medium supplemented with 1% fetal bovine serum (FBS) and incubated with anti-Fc receptor blocking antibody (clone 2.4G2, 10 μg/mL) for 20 minutes at room temperature. Surface staining was performed using cocktails of fluorochrome-conjugated monoclonal antibodies targeting B and T cell populations, including memory B cells and T follicular helper (Tfh) cells. After 30 minutes of incubation at room temperature, cells were washed three times with ice-cold PBS (200 × g, 5 min, 22 °C) and fixed with 1% (w/v) paraformaldehyde in PBS. Samples were acquired on a flow cytometer and analyzed using FlowJo software (Tree Star, Ashland, OR, USA).

### 5.13 ELISpot to identify memory B cells from bone marrow

The ELISpot assay was performed using 96-well MultiScreen filter plates (Millipore, Cat# MSIPS4W10). Wells were initially activated by incubation with 35% (v/v) ethanol for 30 s, followed by two washes with PBS. The plates were then coated with 10 µg/ml of LP1 or LP2 and incubated at 4°C overnight followed by three times washed in PBS. Non-specific binding was prevented by blocking the wells with RPMI 1640 medium supplemented with 10% FBS for 2 h at 37°C under 5%CO_2_. Bone marrow single-cell suspensions were prepared via mechanical disruption, and red blood cells were lysed using ACK lysing buffer (Gibco). Cells were pre-activated for 48 h using the B-Poly-S Polyclonal B Cell Activator (Mabtech Catalog # 3661-1), which included 1 mg/mL of R848 and 1 µg/mL of IL-2. Approximately 106 activated cells were seeded per well in 200 µL of medium and incubated at 37°C in a 5%CO_2_ incubator for an additional 48 h. Spot development was achieved by adding AEC substrate solution for up to 15 min. The reaction was terminated by rinsing the plates under running distilled water. Spots, representing antigen-specific B cells, were enumerated using an ImmunoSpot analyzer.

### 5.14 Quantification of *C. difficile* spores in fecal samples

Feces were collected from mice after *C. difficile* infection on day 0, 1, 3 and 6. 50 gm of the collected feces from each day were dissolved in 500 µl of sterile water and incubated at 4°C overnight to ensure proper dissolving of the samples. Removal of any vegetative cells from the fecal samples was facilitated by redissolving them in 500 µl of absolute ethanol followed by an incubation at room temperature for 1 hour. After removal of ethanol the fecal samples were serially diluted followed by plating them on fructose agar plates supplemented with cefoxitin (8 mg/ml), D-cycloserine (250mg/ml) and taurocholate (0.1% w/v) (52). Inoculated plates were incubated anaerobically at 37°C for 48 hours and the colonies were counted to determine the CFU/gram of feces.

### 5.15 Quantification of *C. difficile* toxin levels in fecal samples

The feces collected after *C. difficile* challenge were dissolved in PBS adulterated with protease inhibitor cocktail at a concentration of 0.1 g/mL. 100 µL of 1 µg/mL of anti-TcdA and anti-TcdB antibodies were coated into each well of the 96-well plate and incubated overnight at 4°C. The plate was washed and blocked with 200 µL of blocking buffer (5% dry milk in PBS) and incubated for 2 hours at 4°C. After washing 100 µL of standards and fecal samples were added to each well and incubated for 1.5 hours at room temperature. Washed plates were then subjected to 100 µL of HRP-chicken anti-*C. difficile* TcdA and TcdB (1:5, 000 dilution) secondary antibodies and incubated 30 min at room temperature. 50µL TMB substrate was added to each well and incubated in dark at room temperature for 20 min. The reaction was stopped with H_2_SO_4,_ and the plate was immediately read at 450 nm a BioGene ^®^ plate reader to determine the absorbance.

### 5.16 Adhesion inhibition of *C. difficile* cells

Human gut epithelium cells (HCT8) grew in 24 well plates till a 95% confluence (approximately 1x10^5^ cells per well) and then moved to the anaerobic chamber for infection. *C. difficile* R20291 vegetative cells were grown till log phase and around 1.5x10^6^ cells were used to infect at a multiplicity of infection (MOI) of 15:1. The *C. difficile* cells were mixed with anti-LP1 and anti-LP2 sera at a dilution of 1/50, 1/100, 1/500 and 1/1000 respectively for incubation at 37°C for 30, before infecting to the HCT8 cells in the plate. After 1.5–2 h of incubation the supernatant was recovered from each well, and the wells were washed twice with 500 µl of PBS. The non-adhered *C. difficile* cells were recovered after a brief centrifugation at 800 x g for 1 min. The recovered non-adhered cells were plated on prereduced BHI plates and colonies were counted to determine the CFU/mL. Experiments were performed in triplicates and preimmunized sera was used as a control. The percentage adherence was calculated by using the following formula: [(initial CFU/mL – eluted CFU/mL)/initial CFU/mL] x 100.

## Data Availability

The original contributions presented in the study are included in the article/**Supplementary Material**. Further inquiries can be directed to the corresponding author.
