# Supplementary material for: Bioinformatic and experimental identification and characterization of Clostridioides difficile lipoproteins as potential vaccine candidates
Source: Front Immunol. 2025 Dec 17;16:1650364. doi: 10.3389/fimmu.2025.1650364 (PMC12753941; doi:10.3389/fimmu.2025.1650364)
Supplement: Supplementary file 1 [file Table1.docx]

**Supplement able 1:** *C. difficile* strains for analysis. Toxin genotypes are reported based on either prior literature source or by BLAST analysis.

| **Ribotype** | **Clade [Reference]** | **Strain** | **Toxins** | **Database** | **Accession number/ Barcode** | **Reference** |
| --- | --- | --- | --- | --- | --- | --- |
| **RT001** | **1 [1, 2]** | **CD105HS12** | **A+B+CDT-** | **GenBank** | **NZ_FMLV00000000.1** | **[3]** |
|  |  | **DSM 1296T** | **A+B+CDT-** | **GenBank** | **CP011968.1** | **[4]** |
|  |  | **C00000193** | **A+B+CDT-** | **EnteroBase** | **CLO_AA3424AA** | **-** |
| **RT002** | **1 [2, 5]** | **S-0253** | **A+B+CDT-** | **GenBank** | **CP076401, CP076402** | **[6]** |
|  |  | **AR-1070** | **A+B+CDT-** | **EnteroBase** | **CLO_CA3086AA** | **[7]** |
|  |  | **CD105HS7** | **A+B+CDT-** | **GenBank** | **NZ_FMLZ00000000.1** | **[3]** |
|  |  | **AR-1074** | **A+B+CDT-** | **GenBank** | **SAMN13029155** | **[7]** |
|  |  | **AR-1084** | **A+B+CDT-** | **GenBank** | **SAMN13029165** | **[7]** |
| **RT003** | **1 [2, 5]** | **VPI 10463** | **A+B+CDT-** | **EnteroBase** | **CLO_AA6882AA** | **[8]** |
|  |  | **C00000562** | **A+B+CDT-** | **EnteroBase** | **CLO_AA3453AA** | **-** |
|  |  | **CD-15-01031** | **A+B+CDT-** | **EnteroBase** | **CLO_BA9410AA** | **-** |
| **RT005 (ST6)** | **1 [2, 9]** | **CD105HS10** | **A+B+CDT-** | **GenBank** | **NZ_FMLY00000000.1** | **[3]** |
|  |  | **C00002831** | **A+B+CDT-** | **EnteroBase** | **CLO_AA2409AA** | **-** |
|  |  | **CD-16-00159** | **A+B+CDT-** | **EnteroBase** | **CLO_BA9401AA** | **-** |
| **RT009** | **1 [2, 5]** | **CD37** | **A-B-CDT-** | **GenBank** | **NZ_AHJJ00000000.1** | **[10]** |
|  |  | **Z31** | **A-B-CDT-** | **GenBank** | **CP013196** | **[11]** |
|  |  | **CD-16-00526** | **A-B-CDT-** | **EnteroBase** | **CLO_BA9556AA** | **-** |
| **RT010 (ST15)** | **1 [1, 2]** | **DSM 29688** | **A-B-CDT-** | **GenBank** | **CP019858.1** | **[12]** |
|  |  | **CD105HS9** | **A-B-CDT-** | **GenBank** | **NZ_FMKY00000000.1** | **[3]** |
|  |  | **NTCD-E4** | **A-B-CDT-** | **GenBank** | **CP118392.1** | **[13]** |
| **RT012** | **1 [2, 5]** | **CD630** | **A+B+CDT-** | **GenBank** | **AM180355.1** | **[14]** |
|  |  | **CD105HS1** | **A+B+CDT-** | **GenBank** | **NZ_FMLF00000000.1** | **[3]** |
|  |  | **6285** | **A+B+CDT-** | **EnteroBase** | **CLO_AA0541AA** | **-** |
| **RT014** | **1 [2, 5]** | **S-0352** | **A+B+CDT-** | **GenBank** | **CP076377** | **[6]** |
|  |  | **AR-1081** | **A+B+CDT-** | **EnteroBase** | **CLO_CA3103AA** | **[7]** |
|  |  | **CD105HS4** | **A+B+CDT-** | **GenBank** | **NZ_FMLB00000000.1** | **[3]** |
|  |  | **AR-1090** | **A+B+CDT-** | **EnteroBase** | **CLO_CA3093AA** | **[7]** |
|  |  | **AR-1091** | **A+B+CDT-** | **GenBank** | **SAMN13029172** | **[7]** |
| **RT015** | **1 [2, 15]** | **173070** | **A-B+CDT-** | **EnteroBase** | **CLO_CA0242AA** | **[16]** |
|  |  | **AR-1069** | **A+B+CDT-** | **EnteroBase** | **CLO_CA3097AA** | **[7]** |
|  |  | **AR-1086** | **A+B+CDT-** | **EnteroBase** | **SAMN13029167** | **[7]** |
| **RT017 (ST37)** | **4 [2, 17]** | **M68** | **A-B+CDT-** | **GenBank** | **NC_017175.1** | **[8]** |
|  |  | **DSM 29627** | **A-B+CDT-** | **GenBank** | **CP016102.1** | **[12]** |
|  |  | **Xy06** | **A-B+CDT-** | **GenBank** | **NZ_JANFNF000000000.1** | **[18]** |
|  |  | **CF5** | **A-B+CDT-** | **GenBank** | **NC_017173.1** | **[17]** |
|  |  | **TGH33** | **A-B+CDT-** | **GenBank** | **JAJNHC000000000** | **[19]** |
|  |  | **TGH51** | **A-B+CDT-** | **GenBank** | **NZ_JAJNHD000000000.1** | **[19]** |
|  |  | **1470** | **A-B+CDT-** | **GenBank** | **NZ_OEZL00000000.1** | **[20]** |
| **RT018 (ST17)** | **1 [2, 21]** | **CD8-15** | **A+B+CDT-** | **GenBank** | **LYDP00000000.1** | **[22]** |
|  |  | **IT1118** | **A+B+CDT-** | **GenBank** | **FAXM00000000** | **[23]** |
|  |  | **CD-16-00005** | **A+B+CDT-** | **EnteroBase** | **CLO_AA7146AA** | **-** |

| **Ribotype** | **Clade [Reference]** | **Strain** | **Toxins** | **Database** | **Accession number/ Barcode** | **References** |
| --- | --- | --- | --- | --- | --- | --- |
| **RT019 (ST67)** | **2 [2, 24]** | **SMG-21-1765** | **A+B+CDT+** | **EnteroBase** | **CLO_FA2592AA_AS** | **-** |
|  |  | **SMG-20-1235** | **A+B+CDT+** | **EnteroBase** | **CLO_EA5278AA_AS** | **-** |
|  |  | **C00002493** | **A+B+CDT+** | **EnteroBase** | **CLO_AA4580AA_AS** | **-** |
|  |  | **AR-1075** | **A+B+CDT+** | **EnteroBase** | **CLO_CA3081AA** | **[7]** |
| **RT020 (ST2)** | **1 [1, 2]** | **AR-1073** | **A+B+CDT-** | **EnteroBase** | **CLO_CA3083AA** | **[7]** |
|  |  | **AR-1080** | **A+B+CDT-** | **EnteroBase** | **CLO_CA3104AA** | **[7]** |
|  |  | **AR-1096** | **A+B+CDT-** | **EnteroBase** | **CLO_CA3087AA** | **[7]** |
| **RT023** | **3 [2, 25]** | **SIRN_ST-001** | **A+B+CDT+** | **EnteroBase** | **CLO_EA5979AA_AS** | **-** |
|  |  | **CD305** | **A+B+CDT-** | **GenBank** | **NZ_JAWXRG000000000.1** | **[25]** |
|  |  | **CD-16-00530** | **A+B+CDT+** | **EnteroBase** | **CLO_DA8015AA_AS** | **-** |
|  |  | **CD-15-00694** | **A+B+CDT+** | **EnteroBase** | **CLO_DA7541AA_AS** | **-** |
|  |  | **DSM 102859** | **A+B+CDT+** | **GenBank** | **NZ_CP020378.1** | **[9]** |
| **RT027 (ST1)** | **2 [2, 5]** | **R20291** | **A+B+CDT+** | **GenBank** | **FN545816.1** | **[26]** |
|  |  | **CD196** | **A+B+CDT+** | **GenBank** | **FN538970.1** | **[27]** |
|  |  | **TGH35** | **A+B+CDT+** | **GenBank** | **JAJNGZ000000000** | **[19]** |
|  |  | **TGH64** | **A+B+CDT+** | **GenBank** | **JAJNHA000000000** | **[19]** |
|  |  | **CD-17-01474** | **A+B+CDT+** | **GenBank** | **NZ_CP026591.1** | **[28]** |
|  |  | **DSM 27638** | **A+B+CDT+** | **GenBank** | **CP011846.1** | **[29]** |
|  |  | **G46** | **A+B+CDT+** | **GenBank** | **NZ_CDND01000001.1** | **[30]** |
|  |  | **TMD0138 (ST97)** | **A+B+CDT+** | **GenBank** | **WUUI00000000.1** | **[31]** |
|  |  | **AR-1067** | **A+B+CDT+** | **EnteroBase** | **CLO_CA3109AA** | **[7]** |
|  |  | **AR-1071** | **A+B+CDT+** | **EnteroBase** | **CLO_CA3085AA** | **[7]** |
|  |  | **AR-1072** | **A+B+CDT+** | **EnteroBase** | **CLO_CA3084AA** | **[7]** |
|  |  | **AR-1076** | **A+B+CDT+** | **GenBank** | **NZ_JADKQV000000000.1** | **[7]** |
|  |  | **AR-1092** | **A+B+CDT+** | **EnteroBase** | **CLO_CA3091AA** | **[7]** |
|  |  | **AR-1095** | **A+B+CDT+** | **EnteroBase** | **CLO_CA3088AA** | **[7]** |
|  |  | **CD105HS8** | **A+B+CDT+** | **GenBank** | **NZ_FMLN00000000.1** | **[3]** |
| **RT031 (ST29)** | **1 [2, 32]** | **CD105HS19** | **A-B-CDT-** | **GenBank** | **NZ_FMLL00000000.1** | **[3]** |
|  |  | **CD-15-00867** | **A-B-CDT-** | **EnteroBase** | **CLO_BA9951AA** | **-** |
| **RT032** | **1 [2]** | **DSM 29637** | **A-B-CDT-** | **GenBank** | **CP016106.1** | **[12]** |
|  |  | **CD-15-01028** | **A-B-CDT-** | **EnteroBase** | **CLO_BA9453AA** | **-** |
|  |  | **SMG-21-2010** | **A-B-CDT-** | **EnteroBase** | **CLO_CA7839AA** | **-** |
| **RT033** | **5 [2, 5]** | **OCD52** | **A-B-CDT+** | **EnteroBase** | **CLO_BA8168AA_AS** | **-** |
|  |  | **IS58** | **A-B-CDT+** | **EnteroBase** | **CLO_AA9965AA_AS** | **[33]** |
|  |  | **RPH0101** | **A-B-CDT+** | **EnteroBase** | **CLO_BA3454AA_AS** | **-** |
| **RT045** | **5 [2, 21]** | **C00002490** | **A+B+CDT+** | **EnteroBase** | **CLO_BA6150AA_AS** | **-** |
|  |  | **CD-16-00514** | **A+B+CDT+** | **EnteroBase** | **CLO_DA7993AA_AS** | **-** |
|  |  | **SIRN_HG-021** | **A+B+CDT+** | **EnteroBase** | **CLO_EA6022AA_AS** | **-** |
| **RT046 (ST35)** | **1 [2, 34]** | **SMG-21-1773** | **A+B+CDT-** | **EnteroBase** | **CLO_CA7611AA** | **-** |
|  |  | **SIRN_HT-039** | **A+B+CDT-** | **EnteroBase** | **CLO_CA2857AA** | **-** |
|  |  | **CD-15-00938** | **A+B+CDT-** | **GenBank** | **DAEMXF000000000.1** | **-** |
| **RT053 (ST63)** | **1 [2, 5, 35]** | **CD-16-00430** | **A+B+CDT-** | **GenBank** | **CLO_BA9519AA** | **-** |
|  |  | **SIRN_ST-026** | **A+B+CDT-** | **EnteroBase** | **CLO_CA2833AA** | **-** |
|  |  | **SMG-22-1463** | **A+B+CDT-** | **EnteroBase** | **CLO_CA9991AA** | **-** |

| **Ribotype** | **Clade [Reference]** | **Strain** | **Toxin** | **Database** | **Accession number/ Barcode** | **References** |
| --- | --- | --- | --- | --- | --- | --- |
| **RT054** | **1 [2, 36]** | **AR-1082** | **A+B+CDT-** | **GenBank** | **SAMN13029163** | **[7]** |
|  |  | **VPT_138** | **A+B+CDT-** | **EnteroBase** | **CLO_CA9950AA** | **-** |
|  |  | **AR-1088** | **A+B+CDT-** | **GenBank** | **SAMN13029169** | **[7]** |
| **RT056 (ST34)** | **1 [2, 37, 38]** | **S-0942** | **A+B+CDT-** | **GenBank** | **CP076376** | **[6]** |
|  |  | **AR-1068** | **A+B+CDT-** | **EnteroBase** | **SAMN13029149** | **[7]** |
|  |  | **AR-1079** | **A+B+CDT-** | **EnteroBase** | **SAMN13029160** | **[7]** |
| **RT059** | **2 [2]** | **8864** | **A-B+CDT-** | **GenBank** | **NZ_OEZE00000000.1** | **[39]** |
| **RT066** | **5 [2, 5]** | **C08-686** | **A+B+CDT+** | **EnteroBase** | **CLO_AA9950AA_AS** | **-** |
|  |  | **CD-16-00440** | **A+B+CDT+** | **EnteroBase** | **CLO_DA7971AA_AS** | **-** |
|  |  | **SMG-21-2330** | **A+B+CDT+** | **EnteroBase** | **CLO_FA3269AA_AS** | **-** |
| **RT078** | **5 [2, 5]** | **TW11-RT078** | **A+B+CDT+** | **GenBank** | **CP035499.1** | **[40]** |
|  |  | **M120** | **A+B+CDT+** | **GenBank** | **CP068555.1** | **[26]** |
|  |  | **R2** | **A+B+CDT+** | **GenBank** | **CP026614.2** | **[41]** |
|  |  | **SUC36** | **A-B+CDT+** | **GenBank** | **NZ_OEZZ00000000.1** | **[16]** |
|  |  | **AR-1077** | **A+B+CDT+** | **EnteroBase** | **CLO_CA3107AA** | **[7]** |
|  |  | **AR-1083** | **A+B+CDT+** | **EnteroBase** | **CLO_CA3101AA** | **[7]** |
|  |  | **CD105HS27** | **A+B+CDT+** | **GenBank** | **NZ_FRET00000000.1** | **[3]** |
|  |  | **CD105HS26** | **A+B+CDT+** | **GenBank** | **NZ_FMLD00000000.1** | **[3]** |
| **RT084** | **1 [2, 42]** | **DSM 28666** | **A-B-CDT-** | **GenBank** | **CP012321.1** | **[12]** |
|  |  | **CD-16-00174** | **A-B-CDT-** | **EnteroBase** | **CLO_BA9789AA** | **-** |
|  |  | **CD-15-00944** | **A-B-CDT-** | **EnteroBase** | **CLO_BA9899AA** | **-** |
| **RT087 (ST46)** | **1 [2, 36, 43]** | **SIRN_HA-012** | **A+B+CDT-** | **EnteroBase** | **CLO_CA2788AA** | **-** |
|  |  | **SMG-20-1333** | **A+B+CDT-** | **EnteroBase** | **CLO_CA2625AA** | **-** |
|  |  | **CD-15-00010** | **A+B+CDT-** | **EnteroBase** | **CLO_AA6357AA** | **-** |
| **RT106** | **1 [2, 36]** | **TGH120** | **A+B+CDT-** | **GenBank** | **JAJNHB000000000** | **[19]** |
|  |  | **DH/NAP11/106/ST-42** | **A+B+CDT-** | **GenBank** | **CP022524.1** | **[44]** |
|  |  | **C00000224** | **A+B+CDT+** | **EnteroBase** | **CLO_AA4561AA_AS** | **-** |
|  |  | **CD-16-00068** | **A+B+CDT-** | **EnteroBase** | **CLO_DA8040AA_AS** | **-** |
|  |  | **AR-1078** | **A+B+CDT-** | **EnteroBase** | **CLO_CA3106AA** | **[7]** |
|  |  | **AR-1085** | **A+B+CDT-** | **EnteroBase** | **CLO_CA3099AA** | **[7]** |
|  |  | **AR-1087** | **A+B+CDT-** | **EnteroBase** | **CLO_CA3096AA** | **[7]** |
|  |  | **AR-1089** | **A+B+CDT-** | **EnteroBase** | **CLO_CA3094AA** | **[7]** |
|  |  | **AR-1093** | **A+B+CDT-** | **EnteroBase** | **CLO_CA3090AA** | **[7]** |
| **RT125** | **C-III [45]** | **HGP05 (ST848)** | **A-B-CDT-** | **GenBank** | **CP103977** | **[46]** |
|  |  | **C00006475** | **A-B-CDT-** | **EnteroBase** | **CLO_AA4820AA** | **-** |
| **RT126** | **5 [2, 5]** | **6058625** | **A+B+CDT+** | **EnteroBase** | **CLO_BA0391AA_AS** | **-** |
|  |  | **CD-16-00082** | **A+B+CDT+** | **EnteroBase** | **CLO_AA7075AA_AS** | **-** |
|  |  | **DSM 29020** | **A+B+CDT+** | **GenBank** | **CP012325.1** | **[12]** |
| **RT220 (ST3)** | **1 [2, 38, 47]** | **CD105HS22** | **A+B+CDT-** | **GenBank** | **NZ_FMLJ00000000.1** | **[3]** |
|  |  | **CD105HS6** | **A+B+CDT-** | **GenBank** | **NZ_FMLI00000000.1** | **[3]** |
|  |  | **CD-16-00120** | **A+B+CDT-** | **EnteroBase** | **CLO_BA9841AA** | **-** |
| **RT244** | **2 [2, 48]** | **C00009691** | **A+B+CDT+** | **EnteroBase** | **CLO_AA0004AA_AS** | **-** |
|  |  | **C00009694** | **A+B+CDT+** | **EnteroBase** | **CLO_AA0003AA_AS** | **-** |
|  |  | **C00009695** | **A+B+CDT+** | **EnteroBase** | **CLO_AA0002AA_AS** | **-** |

| **Ribotype** | **Clade [Reference]** | **Strain** | **Toxin Genotype** | **Database** | **Accession number/ Barcode** | **References** |
| --- | --- | --- | --- | --- | --- | --- |
| **RT255 (ST34)** | **1 [2, 43, 49]** | **Mta-79** | **A+B+CDT-** | **GenBank** | **CP042267** | **[49]** |
| **RT871** | **3 [2, 50]** | **LC693** | **A+B+CDT+** | **GenBank** | **NCXL00000000.1** | **[50, 51]** |
| **SLO 091** | **4 [2]** | **DSM 28669** | **A-B-CDT-** | **GenBank** | **CP012323** | **[12]** |
| **SLO101** | **5 [2]** | **ES130** | **A-B+CDT-** | **GenBank** | **NZ_OEZV00000000.1** | **[16]** |
| **SLO 235** | **4 [2]** | **DSM 29629** | **A-B-CDT-** | **GenBank** | **CP016104** | **[12]** |
| **SLO 237** | **4 [2]** | **DSM 28670** | **A-B-CDT-** | **GenBank** | **CP012312** | **[12]** |
| **SKO098** | **5 [2]** | **WA151** | **A-B+CDT-** | **GenBank** | **NZ_OEZY00000000.1** | **[16]** |
| **ST1** | **2 [2, 5]** | **TGH29** | **A+B+CDT+** | **GenBank** | **JAPKMB000000000.1** | **[52]** |
|  |  | **R0104a** | **A+B+CDT+** | **GenBank** | **CP025044** | **[53]** |
| **ST2** | **1 [2, 5]** | **W0022a** | **A+B+CDT-** | **GenBank** | **CP025045** | **[53]** |
| **ST3** | **1 [2, 38]** | **CCUG37785** | **A-B-CDT-** | **GenBank** | **NZ_JAGKRT000000000.1** | **[54]** |
| **ST8** | **1 [2, 5]** | **W0003a** | **A+B+CDT-** | **GenBank** | **CP025047.1** | **[53]** |
| **ST11** | **5 [2, 5]** | **TGH79** | **A+B+CDT+** | **GenBank** | **NZ_JAPKMA000000000.1** | **[52]** |
|  |  | **2301802** | **A+B+CDT+** | **GenBank** | **JBEJVX000000000.1** | **[55]** |
| **ST15** | **1 [2, 38]** | **TGH132** | **A-B-CDT-** | **GenBank** | **JAPKMC000000000.1** | **[52]** |
| **ST23** | **4 [2, 56]** | **KS145** | **A-B-CDT-** | **GenBank** | **JARJHO000000000.1** | **[57]** |
| **ST35** | **1[2, 38]** | **TGH91** | **A+B+CDT-** | **GenBank** | **JAPKLZ000000000.1** | **[52]** |
| **ST42** | **1 [2, 38]** | **W0023a** | **A+B+CDT-** | **GenBank** | **CP025045.1** | **[53]** |
| **ST99** | **1 [2, 43]** | **2301801** | **A+B+CDT+** | **GenBank** | **JBEJVW000000000.1** | **[55]** |
| **ST109** | **4 [2, 56]** | **TGH114** | **A-B-CDT-** | **GenBank** | **JAPKMD000000000.1** | **[52]** |
| **ST340** | **C-III [58]** | **MA_2** | **A-B-CDT-** | **GenBank** | **CP129431.1** | **[59]** |

**Supplementary Figure Legend**

**Figure S1:** Immune simulation in CImmSim server, the cytotoxic T cells population increment after immunization with LP1 (A) and LP2 (B); the helper T cells population surge after immunization with LP1 (C) and LP2 (D)

**Figure S2:** Immune simulation in CImmSim server, showing the activation of Macrophages after immunizations with LP1 (A) and LP2 (D), activation of Dendritic cells by LP1 (B) and LP2 (E), and increase of Natural Killer cell population by LP1 (C) and LP2 (F).

**Figure S1:**

**
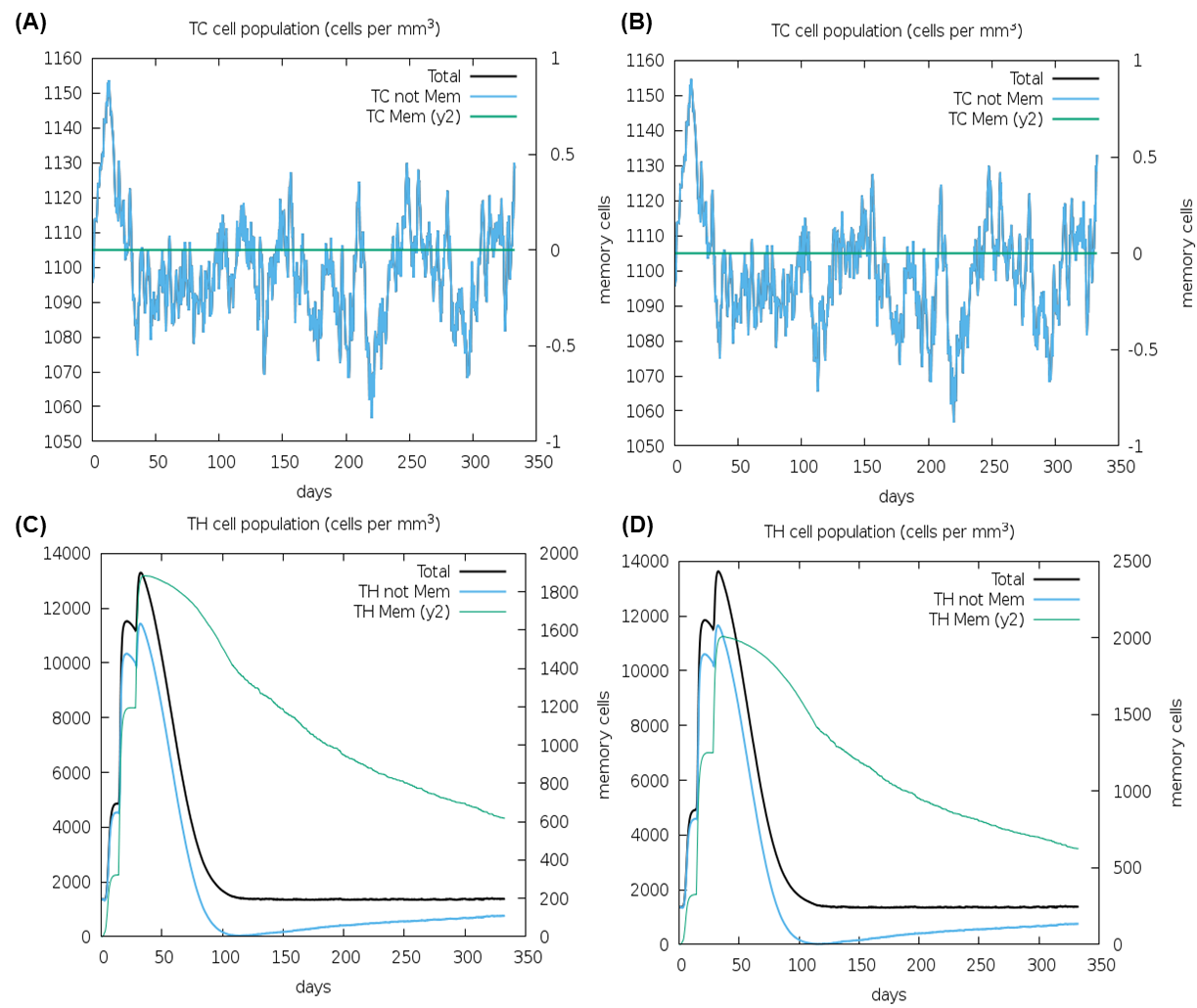
**

**Figure S2:**


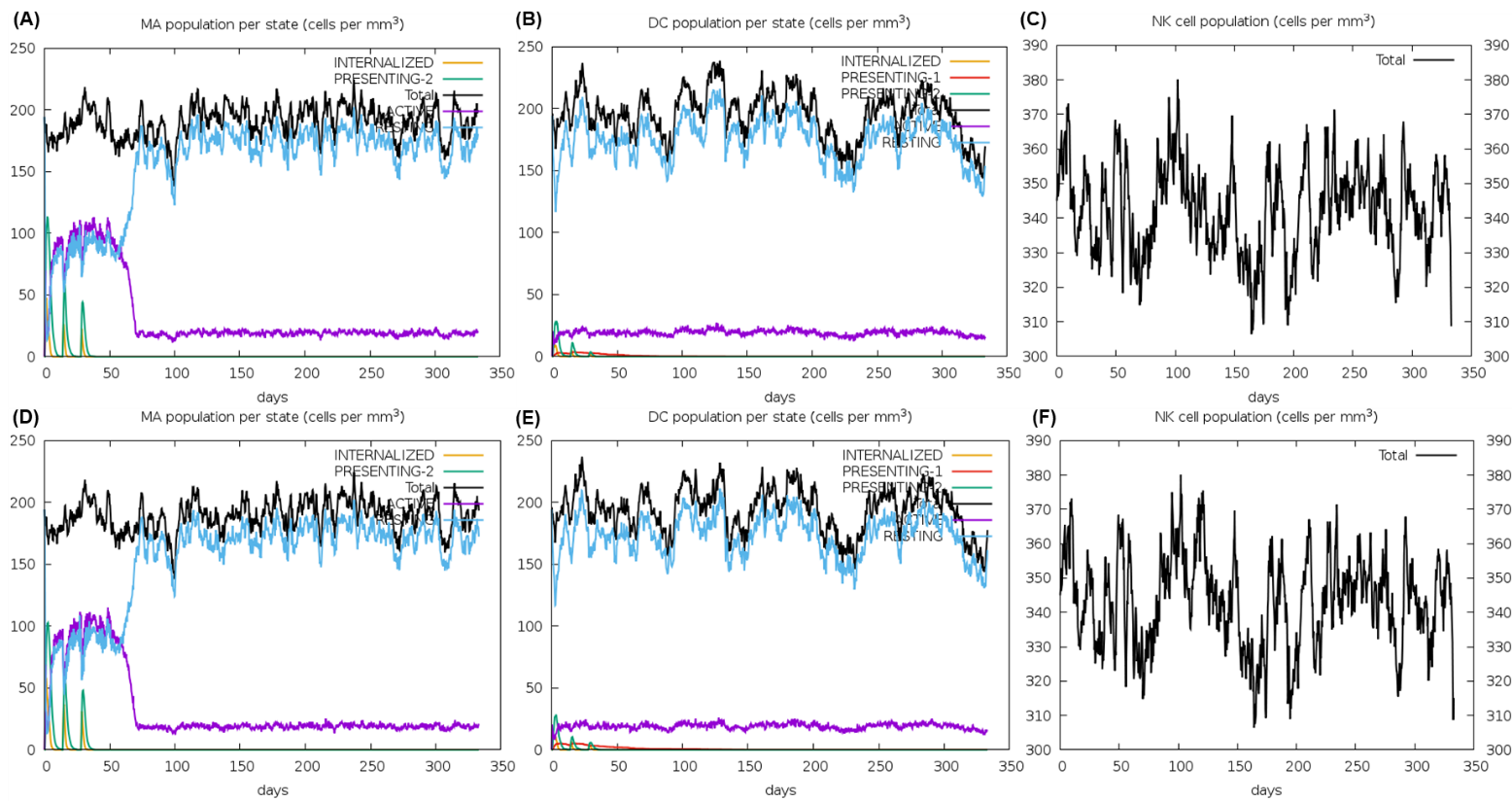


**Supplementary References:**

1. Blau, K., et al., *Clostridioides difficile from fecally contaminated environmental sources: resistance and genetic relatedness from a molecular epidemiological perspective.* Microorganisms, 2023. **11**(10): p. 2497.

2. Jolley, K.A., J.E. Bray, and M.C.J. Maiden, *Open-access bacterial population genomics: BIGSdb software, the PubMLST.org website and their applications.* Wellcome Open Res, 2018. **3**: p. 124.

3. Hargreaves, K.R., et al., *As Clear as Mud? Determining the Diversity and Prevalence of Prophages in the Draft Genomes of Estuarine Isolates of Clostridium difficile.* Genome Biology and Evolution, 2015. **7**(7): p. 1842-1855.

4. Riedel, T., et al., *Complete Genome Sequence of the Clostridium difficile Type Strain DSM 1296T.* Genome Announcements, 2015. **3**(5): p. 10.1128/genomea.01186-15.

5. Collins, J., et al., *Dietary trehalose enhances virulence of epidemic Clostridium difficile.* Nature, 2018. **553**(7688): p. 291-294.

6. O’Grady, K., V. Riley Thomas, and R. Knight Daniel, *Complete Genome Assemblies of Three Highly Prevalent, Toxigenic Clostridioides difficile Strains Causing Health Care-Associated Infections in Australia.* Microbiology Resource Announcements, 2021. **10**(31): p. 10.1128/mra.00599-21.

7. Paulick, A., et al., *Characterization of Clostridioides difficile Isolates Available through the CDC & FDA Antibiotic Resistance Isolate Bank.* Microbiology Resource Announcements, 2021. **10**(1): p. 10.1128/mra.01011-20.

8. Cairns, M., et al., *Genomic epidemiology of a protracted hospital outbreak caused by a toxin A-negative Clostridium difficile sublineage PCR ribotype 017 strain in London, England.* Journal of clinical microbiology, 2015. **53**(10): p. 3141-3147.

9. Rupnik, M., et al., *Distribution of Clostridioides difficile ribotypes and sequence types across humans, animals and food in 13 European countries.* Emerg Microbes Infect, 2024. **13**(1): p. 2427804.

10. Brouwer, M.S., et al., *Genetic organisation, mobility and predicted functions of genes on integrated, mobile genetic elements in sequenced strains of Clostridium difficile.* PloS one, 2011. **6**(8): p. e23014.

11. Pereira, F.L., et al., *Complete genome sequence of Peptoclostridium difficile strain Z31.* Gut pathogens, 2016. **8**: p. 1-7.

12. Riedel, T., et al., *High metabolic versatility of different toxigenic and non-toxigenic Clostridioides difficile isolates.* International Journal of Medical Microbiology, 2017. **307**(6): p. 311-320.

13. Etifa, P., et al., *Non-toxigenic Clostridioides difficile strain E4 (NTCD-E4) prevents establishment of primary C. difficile infection by epidemic PCR ribotype 027 in an in vitro human gut model.* Antibiotics, 2023. **12**(3): p. 435.

14. Sebaihia, M., et al., *The multidrug-resistant human pathogen Clostridium difficile has a highly mobile, mosaic genome.* Nature genetics, 2006. **38**(7): p. 779-786.

15. Abad-Fau, A., et al., *Update on commonly used molecular typing methods for Clostridioides difficile.* Microorganisms, 2023. **11**(7): p. 1752.

16. Janezic, S., et al., *Comparative genomics of Clostridioides difficile toxinotypes identifies module-based toxin gene evolution.* Microbial genomics, 2020. **6**(10): p. e000449.

17. Imwattana, K., et al., *Clostridium difficile ribotype 017–characterization, evolution and epidemiology of the dominant strain in Asia.* Emerging microbes & infections, 2019. **8**(1): p. 796-807.

18. Li, C., et al., *Genomic and phenotypic characterization of a Clostridioides difficile strain of the epidemic ST37 type from China.* Frontiers in Cellular and Infection Microbiology, 2024. **14**: p. 1412408.

19. Wickramage, I., et al., *The vanRCd mutation 343A> G, resulting in a Thr115Ala substitution, is associated with an elevated minimum inhibitory concentration (MIC) of vancomycin in clostridioides difficile clinical isolates from florida.* Microbiology Spectrum, 2023. **11**(3): p. e03777-22.

20. Depitre, C., et al., *Serogroup F strains of Clostridium difficile produce toxin B but not toxin A.* Journal of medical microbiology, 1993. **38**(6): p. 434-441.

21. Knight, D.R., et al., *Diversity and evolution in the genome of Clostridium difficile.* Clinical microbiology reviews, 2015. **28**(3): p. 721-741.

22. Riccobono, E., et al., *Draft Genome Sequence of Clostridium difficile Belonging to Ribotype 018 and Sequence Type 17.* Genome Announcements, 2016. **4**(5): p. 10.1128/genomea.00907-16.

23. Wasels, F., F. Barbanti, and P. Spigaglia, *Draft Genome Sequence of Clostridium difficile Strain IT1118, an Epidemic Isolate Belonging to the Emerging PCR Ribotype 018.* Genome Announcements, 2016. **4**(4): p. 10.1128/genomea.00717-16.

24. Saito, R., et al., *Hypervirulent clade 2, ribotype 019/sequence type 67 Clostridioides difficile strain from Japan.* Gut pathogens, 2019. **11**: p. 1-7.

25. Shaw, H.A., et al., *The recent emergence of a highly related virulent Clostridium difficile clade with unique characteristics.* Clinical Microbiology and Infection, 2020. **26**(4): p. 492-498.

26. He, M., et al., *Evolutionary dynamics of Clostridium difficile over short and long time scales.* Proceedings of the National Academy of Sciences, 2010. **107**(16): p. 7527-7532.

27. Tasteyre, A., et al., *A Clostridium difficile gene encoding flagellin.* Microbiology, 2000. **146**(4): p. 957-966.

28. Steglich, M., et al., *Convergent loss of ABC transporter genes from Clostridioides difficile genomes is associated with impaired tyrosine uptake and p-cresol production.* Frontiers in microbiology, 2018. **9**: p. 901.

29. Groß, U., et al., *Comparative genome and phenotypic analysis of three Clostridioides difficile strains isolated from a single patient provide insight into multiple infection of C. difficile.* BMC genomics, 2018. **19**(1): p. 1-14.

30. Gaulton, T., et al., *Complete Genome Sequence of the Hypervirulent Bacterium Clostridium difficile Strain G46, Ribotype 027.* Genome Announcements, 2015. **3**(2): p. 10.1128/genomea.00073-15.

31. Usui, Y., et al., *Draft Genome Sequence of a Clostridioides difficile Sequence Type 97 Strain Belonging to Hypervirulent Clade 2.* Microbiology Resource Announcements, 2020. **9**(14): p. 10.1128/mra.00245-20.

32. Roy Chowdhury, P., et al., *Comparative genomic analysis of toxin-negative strains of Clostridium difficile from humans and animals with symptoms of gastrointestinal disease.* BMC microbiology, 2016. **16**: p. 1-13.

33. Stubbs, S., et al., *Production of actin-specific ADP-ribosyltransferase (binary toxin) by strains of Clostridium difficile.* FEMS microbiology letters, 2000. **186**(2): p. 307-312.

34. Cerri, F.M., et al., *Fecal shedding of Clostridioides difficile in calves in Sao Paulo state, Brazil.* Anaerobe, 2024. **88**: p. 102861.

35. Williamson, C.H., et al., *A global to local genomics analysis of Clostridioides difficile ST1/RT027 identifies cryptic transmission events in a northern Arizona healthcare network.* Microbial genomics, 2019. **5**(7): p. e000271.

36. Knetsch, C.W., et al., *Comparative analysis of an expanded Clostridium difficile reference strain collection reveals genetic diversity and evolution through six lineages.* Infection, Genetics and Evolution, 2012. **12**(7): p. 1577-1585.

37. O’grady, K., et al., *Defining the phylogenetics and resistome of the major Clostridioides difficile ribotypes circulating in Australia.* Microbial genomics, 2024. **10**(5): p. 001232.

38. Knight, D.R., et al., *Major genetic discontinuity and novel toxigenic species in Clostridioides difficile taxonomy.* elife, 2021. **10**: p. e64325.

39. Soehn, F., et al., *Genetic rearrangements in the pathogenicity locus of Clostridium difficile strain 8864–implications for transcription, expression and enzymatic activity of toxins A and B.* Molecular and General Genetics MGG, 1998. **258**(3): p. 222-232.

40. Fatoba, A.J., D.O. Fatoba, and S.O. Babalola, *Pangenome and subtractive genomic analysis of Clostridioides difficile reveals putative drug targets.* Journal of Proteins and Proteomics, 2022. **13**(4): p. 247-256.

41. Kumar, A., et al., *Complete genome sequences of historic Clostridioides difficile food-dwelling ribotype 078 strains in Canada identical to that of the historic human clinical strain M120 in the United Kingdom.* Microbiology Resource Announcements, 2018. **7**(12): p. 10.1128/mra. 00853-18.

42. Ngbede, E.O., et al., *Clostridioides difficile recovered from hospital patients, livestock and dogs in Nigeria share near-identical genome sequences.* Microbial Genomics, 2025. **11**(1): p. 001342.

43. Stabler, R.A., et al., *Macro and micro diversity of Clostridium difficile isolates from diverse sources and geographical locations.* PloS one, 2012. **7**(3): p. e31559.

44. Ozer, E.A., et al., *Complete genome sequence of Clostridioides difficile epidemic strain DH/NAP11/106/ST-42, isolated from stool from a pediatric patient with diarrhea.* Genome Announcements, 2017. **5**(38): p. 10.1128/genomea. 00923-17.

45. Shivaperumal, N., et al., *Esculin hydrolysis negative and TcdA‐only producing strains of Clostridium (Clostridioides) difficile from the environment in Western Australia.* Journal of applied microbiology, 2022. **133**(3): p. 1183-1196.

46. Shivaperumal, N., et al., *Complete genome sequences of evolutionary clade C-III strains of Clostridioides (Clostridium) difficile isolated from the environment in Western Australia.* Microbiology resource announcements, 2023. **12**(5): p. e00239-23.

47. Zhang, W.-Z., et al., *The molecular characters and antibiotic resistance of Clostridioides difficile from economic animals in China.* BMC microbiology, 2020. **20**: p. 1-7.

48. Lim, S.K., et al., *Emergence of a ribotype 244 strain of Clostridium difficile associated with severe disease and related to the epidemic ribotype 027 strain.* Clinical Infectious Diseases, 2014. **58**(12): p. 1723-1730.

49. Spinler Jennifer, K., et al., *Complete Genome Sequence of Clostridioides difficile Ribotype 255 Strain Mta-79, Assembled Using Oxford Nanopore and Illumina Sequencing.* Microbiology Resource Announcements, 2019. **8**(42): p. 10.1128/mra.00935-19.

50. Li, C., et al., *Characterization of the virulence of a non-RT027, non-RT078 and binary toxin-positive Clostridium difficile strain associated with severe diarrhea.* Emerging Microbes & Infections, 2018. **7**(1): p. 1-11.

51. Peng, Z., et al., *Genome characterization of a novel binary toxin-positive strain of Clostridium difficile and comparison with the epidemic 027 and 078 strains.* Gut Pathogens, 2017. **9**: p. 1-16.

52. Wickramage, I., et al., *Draft Genome Sequences and Genome Characterization of Three Toxigenic and Two Nontoxigenic Clostridioides difficile Clinical Isolates from Florida, USA.* Microbiology Resource Announcements, 2023. **12**(5): p. e00151-23.

53. Yin, C., et al., *Complete Genome Sequences of Four Toxigenic Clostridium difficile Clinical Isolates from Patients of the Lower Hudson Valley, New York, USA.* Genome Announcements, 2018. **6**(4): p. 10.1128/genomea.01537-17.

54. Wang, S., et al., *Genomic and Phenotypic Characterization of the Nontoxigenic Clostridioides difficile Strain CCUG37785 and demonstration of its therapeutic potential for the prevention of C. difficile Infection.* Microbiology Spectrum, 2022. **10**(2): p. e01788-21.

55. Lu, X., Y. Feng, and X. Wang, *Draft genome sequences of two distinct Clostridioides difficile isolates coinfecting a patient.* Microbiology Resource Announcements, 2024. **0**(0): p. e01017-24.

56. Dingle, K.E., et al., *Evolutionary history of the Clostridium difficile pathogenicity locus.* Genome biology and evolution, 2014. **6**(1): p. 36-52.

57. Yaikhan, T., et al., *Genome Sequence of a Clostridioides difficile Strain Isolated from Feces from a Patient in Southern Thailand.* Microbiology Resource Announcements, 2023. **12**(7): p. e00455-23.

58. Schüler, M.A., et al., *Culture-independent detection of low-abundant Clostridioides difficile in environmental DNA via PCR.* Applied and Environmental Microbiology, 2024. **90**(3): p. e01278-23.

59. Schüler Miriam, A., R. Daniel, and A. Poehlein, *Complete genome sequence of a Clostridioides difficile cryptic C-III strain isolated from horse feces.* Microbiology Resource Announcements, 2023. **12**(12): p. e00781-23.
